# Balanced Expression of the Diiron Oxygenase BioE Is Essential for Biotin Homeostasis in *Elizabethkingia meningoseptica*

**DOI:** 10.1002/advs.202510491

**Published:** 2025-12-12

**Authors:** Meng Zhang, Ying‐ying Fu, Xiaoqiang Yang, Qiuying Qin, Xinyu Su, Jiaming Fang, Yanhua Kang, Qingwen He, Zhi Ruan, Yongchang Xu

**Affiliations:** ^1^ Zhejiang Key Laboratory of Medical Epigenetics Department of Immunology and Pathogen Biology School of Basic Medical Sciences Hangzhou Normal University Hangzhou Zhejiang 311121 China; ^2^ Department of Clinical Laboratory Shanghai East Hospital Tongji University School of Medicine Shanghai 200120 China; ^3^ Department of Clinical Laboratory Sir Run Run Shaw Hospital Zhejiang University School of Medicine Hangzhou Zhejiang 310016 China

**Keywords:** antimicrobial target, BioE diiron oxygenase, BioL regulator, biotin biosynthesis pathway, fitness cost

## Abstract

Biotin is an essential cofactor for central metabolic pathways in all organisms. The newly identified BioE‐BioL module constitutes a new biotin biosynthesis pathway, yet its mechanisms remain incompletely characterized. Phylogenetic analyses reveal widespread distribution of the *bioE*, including obligate intracellular *Chlamydia*, despite the genus lacking its cognate repressor BioL. Structural modeling and biochemical characterization of *Elizabethkingia meningoseptica* BioE (EmBioE) and *Chlamydia psittaci* BioE (CpBioE) reveal a conserved diiron oxygenase catalytic core but divergent oligomeric structure state and substrate preferences. EmBioE forms a homodimer capable of recognizing both long‐chain acyl‐ACP and acyl‐CoA, whereas CpBioE functions as a monomer restricted to acyl‐ACP. Heterologous overexpression of EmBioE, but not CpBioE, induces a fitness cost in *Escherichia coli*. Genetic ablation of *bioL* leads to biotin auxotrophy in *Elizabethkingia*, mainly attributed to the unregulated EmBioE pathway exhausting long‐chain fatty acids and depleting ATP/SAM metabolic pools. This highlights EmBioE's biphasic role: initiating biotin synthesis to sustain viability while inducing stress upon overexpression, requiring BioL regulation for metabolic homeostasis. Virtual screening uncovers compound 466982 as a selective BioE inhibitor with dose‐dependent antibacterial activity against *Elizabethkingia*. Balanced BioE expression is critical for bacterial viability, positioning BioE as a druggable target for antimicrobial discovery against multidrug‐resistant pathogens.

## Graphical abstract

BioE is a new diiron oxygenase that catalyzes the conversion of long‐chain acyl groups into pimeloyl thioester, initiating biotin synthesis. The overexpression of EmBioE disrupts lipid metabolic homeostasis, requiring repressor BioL to maintain a balance between long‐chain fatty acids and biotin synthesis. The BioE‐targeting inhibitor 466982 blocks biotin synthesis, emerging as a potential antibacterial agent against *Elizabethkingia*.

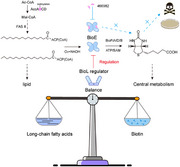

## 1 Introduction

Biotin, a derivative of fatty acid, is a water‐soluble indispensable vitamin H that serves as a prosthetic group for a multitude of metabolic enzymes catalyzing carboxyl‐transfer reactions.^[^ ^1^, ^2^, ^3^ ^]^ It plays a crucial role in lipid biosynthesis, gluconeogenesis, and amino acid catabolism through post‐translational modification of key enzymes. The biotinylation of proteins, such as acetyl‐CoA carboxylase (AccB), is catalyzed by biotin ligase and is essential for initiating fatty acid synthesis.^[^ ^4^, ^5^ ^]^ Moreover, Biotin is broadly applied in pharmaceutical, cosmetics, food additives, and livestock industries, with a world market of 10–30 tons annually.^[^ ^6^, ^7^ ^]^ Given the environmental toll of current chemical synthesis methods, there is an imperative need to develop more eco‐friendly biosynthetic alternatives. Unlike certain plants and bacteria, mammals are incapable of *de novo* biotin synthesis and are entirely dependent on exogenous sources. Consequently, enzymes in the biotin synthesis pathway are potential targets for the development of new antibiotics. This is underscored by the validation of biotin synthetic genes as effective antibacterial targets in pathogens such as *Mycobacterium tuberculosis* (*M. tuberculosis*), *Francisella tularensis* (*F. tularensis*), *Acinetobacter baumannii* (*A. baumannii*), *Klebsiella pneumoniae* (*K. pneumoniae*), and *Pseudomonas aeruginosa* (*P. aeruginosa*).^[^ ^8^, ^9^, ^10^, ^11^, ^12^, ^13^ ^]^

The biotin biosynthetic pathway is divided into an early stage, involving the formation of pimeloyl moiety, and a late stage, which includes the assembly of the biotin ring.^[^ ^14^ ^]^ Pioneering research has elucidated the late stages of this pathway, characterized by the conserved *bioF*, *bioA*, *bioD*, and *bioB* genes across bacterial species.^[^ ^15^ ^]^ These genes encode four highly conserved enzymes responsible for the conversion of pimeloyl group into biotin: 8‐amino‐7‐oxononanoate synthase (AONS, encoded by *bioF*), 7,8‐diaminopelargonic acid synthase (DANS, encoded by *bioA*), dethiobiotin synthetase (DTBS, encoded by *bioD*), and biotin synthase (BS, encoded by *bioB*). These enzymes orchestrate a series of ATP‐ and S‐adenosylmethionine (SAM)‐dependent reactions to forge the complex architecture of biotin, highlighting the sophistication of this biosynthetic cascade.^[^ ^15^, ^16^ ^]^ Sakaki et al. found the BioU, a suicide enzyme in Cyanobacteria that functionally complements BioA. Biochemical characterizations reveal BioU as a novel dehydrogenase that catalyzes three sequential reactions: i) formation of a covalent BioU‐DAN conjugate via NAD(P)H‐dependent condensation with 8‐amino‐7‐oxononanoate (AON) at the ε‐amino group of Lys124; ii) carboxylation of this conjugate to generate BioU‐DAN‐carbamic acid; and iii) NAD(P)^+^‐driven release of DAN‐carbamic acid. Mechanistically, BioU acts as a single‐turnover enzyme, as the catalytic Lys124 residue is irreversibly modified during the reaction cycle.^[^ ^17^ ^]^ Recently, mycobacterial biotin biosynthesis was shown to require BsaP, an accessory factor essential for *Mycobacterium* BioB to catalyze the conversion of dethiobiotin (DTB) to biotin. BsaP harbors an atypical Fe‐S cluster that facilitates sulfur insertion into DTB, underscoring the discovery of mechanistic diversity in the later steps of biotin formation.^[^ ^5^ ^]^

In the landscape of biotin biosynthesis, the *de novo* generation of the pimeloyl group, critical as a biotin precursor in the form of pimeloyl‐CoA or pimeloyl‐acyl carrier protein (ACP), exhibits striking interspecies diversity.^[^ ^18^ ^]^ The diversity is defined by three predominant pathways: the archetypal BioC‐BioH pathway characterized in *Escherichia coli* (*E. coli*), the BioI‐BioW pathway exemplified by *Bacillus subtilis* (*B. subtilis*), and the BioZ pathway identified in *Agrobacterium tumefaciens* (*A. tumefaciens*).^[^ ^18^, ^19^ ^]^ BioC, an SAM dependent methyltransferase, initiates the biosynthetic cascade by methylating malonyl‐ACP, setting the stage for subsequent elongation through fatty acid synthase cycles to yield methyl‐pimeloyl‐ACP.^[^ ^20^ ^]^ The esterase BioH then demethylates this intermediate, releasing pimeloyl‐ACP.^[^ ^21^ ^]^ The diversity of BioH is echoed in its homologs, such as BioG in *Haemophilus influenzae* (*H. influenzae*), BioK in *Synechococcus spp*, BioJ in *F. tularensis*, and BioV in *Helicobacter pylori* (*H. pylori*), with *Mycobacterium smegmatis* (*M. smegmatis*) harboring three BioH isoenzymes.^[^ ^22^, ^23^, ^24^, ^25^, ^26^ ^]^ In *B. subtilis*, BioI and BioW represent parallel pathways; BioI, a heme‐dependent cytochrome P450 enzyme, orchestrates the *de novo* synthesis of pimeloyl‐ACP from long‐chain acyl‐ACPs.^[^ ^27^, ^28^ ^]^ The BioI is unique to *B. subtilis*, and bypasses the BioC‐BioH pathway in *E. coli*.^[^ ^29^ ^]^ BioW is an ATP‐dependent acyl‐CoA synthetase that selectively synthesizes pimeloyl‐CoA from free pimelic acid with a stringent substrate preference.^[^ ^30^, ^31^ ^]^ Interestingly, in contrast to *E. coli* BioF(EcBioF), which accommodates both pimeloyl‐ACP and pimeloyl‐CoA, *B. subtilis* BioF(BsBioF) specifically employs pimeloyl‐CoA as a bona fide precursor for biotin synthesis.^[^ ^32^ ^]^ Given that BioI exclusively utilizes long‐chain acyl‐ACP (not acyl‐CoA) as a substrate and generates pimeloyl‐ACP, a product unrecognized by BsBioF, BioI is rendered functionally redundant in *B. subtilis*.^[^ ^32^ ^]^ The non‐canonical BioZ pathway, prevalent in α‐proteobacteria like *A. tumefaciens*, features BioZ, a β‐ketoacyl‐ACP synthase III family enzyme, which catalyzes the condensation of glutaryl‐CoA with malonyl‐ACP to produce 5′‐keto‐pimeloyl‐ACP. This intermediate engages in the type II fatty acid synthesis pathway, initiating pimeloyl‐ACP formation.^[^ ^33^, ^34^, ^35^, ^36^ ^]^ Our team recently identified a new BioE‐dependent biotin biosynthesis pathway in emerging multidrug‐resistant *Elizabethkingia* and *Chryseobacterium*, representing the fourth characterized biotin synthesis route. BioE, a non‐heme Fe‐dependent oxygenase, catalyzes the C7‐C8 cleavage of long‐chain acyl groups to generate pimeloyl‐ACP or pimeloyl‐CoA.^[^ ^37^ ^]^ This pathway is regulated by the repressor BioL, a MocR‐family transcriptional regulator that differs from BirA (which directly senses biotin concentration).^[^ ^11^, ^37^, ^38^ ^]^ Unlike BirA, BioL negatively regulates biotin synthesis by sensing the intermediate 8‐amino‐7‐oxononanoate(AON), adding a layer of metabolic control distinct from canonical biotin feedback mechanisms.^[37,^ ^39^ ^]^ However, numerous questions remain regarding the mechanisms underlying the regulation of BioE‐BioL and its functional interplay.

In this study, we performed a systematic genetic analysis of *bioE*, revealing its presence in diverse bacterial families, including *Chlamydia*. A striking paradox emerged within *Chlamydia*, although *bioE* is highly conserved across most *Chlamydia* species, its cognate regulatory *bioL* is absent from this genus. Structural and biochemical comparisons between EmBioE and CpBioE revealed conserved diiron active sites but divergent oligomerization states; EmBioE functions as a homodimer, whereas CpBioE acts as a monomer. Mechanistically, EmBioE exhibits promiscuous substrate binding to both long‐chain acyl‐ACP and acyl‐CoA, while CpBioE is strictly specific to acyl‐ACP. Genetic studies in *Elizabethkingia* showed that Δ*bioL* mutants displayed biotin auxotrophy, a phenotype absent in *Chlamydia* despite its natural *bioL* deficiency. Metabolic analyses revealed significantly reduced intracellular ATP/SAM levels in Δ*bioL* strains, directly linking BioL‐mediated regulation to central metabolic homeostasis. Heterologous overexpression of EmBioE, but not CpBioE, induced growth arrest in *E. coli*, a phenotype rescued by exogenous long‐chain fatty acid supplementation. This emphasizes the critical need for balanced EmBioE expression to maintain bacterial viability. Structure‐based virtual screening identified small molecule 422689 as a potent EmBioE inhibitor with dose‐dependent antibacterial activity against *Elizabethkingia*. Collectively, our findings establish the broad phylogenetic distribution of the BioE pathway across bacterial pathogens and validate its therapeutic potential as a druggable target for treating infections caused by BioE‐expressing pathogens.

## 2 Results

### 2.1 Wide Distribution of the BioE Biotin Biosynthesis Pathway

**Figure 1:**
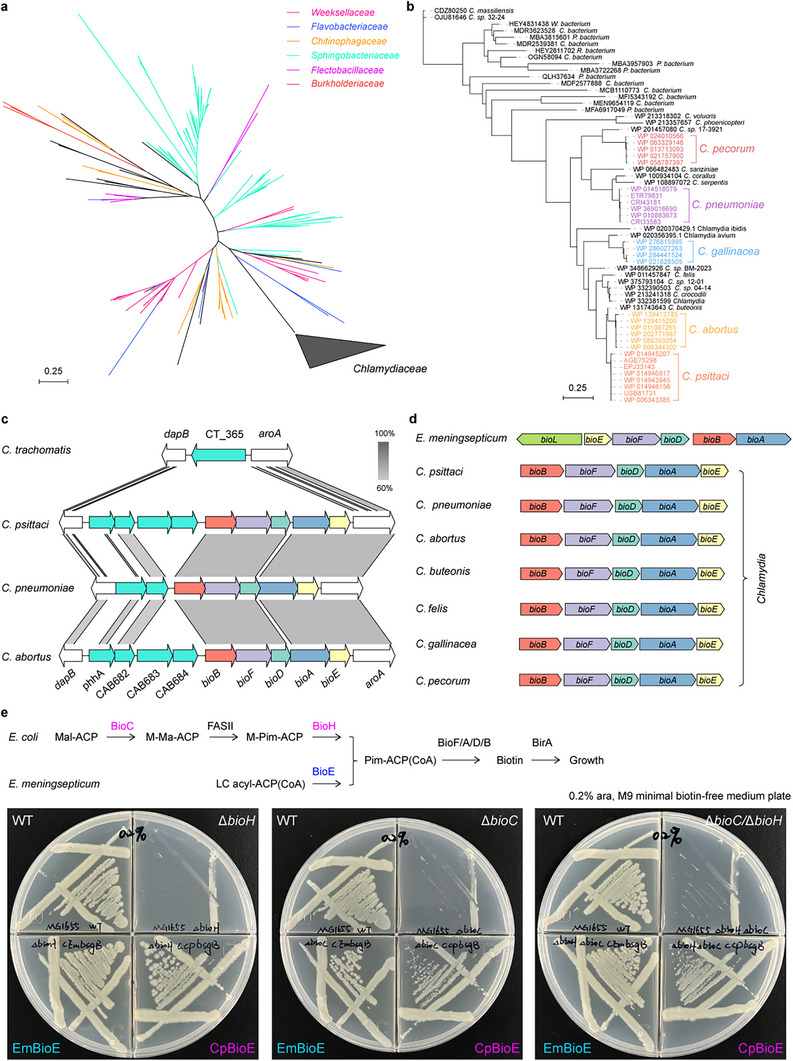
Widespread distribution and genetic diversity of the *bioE* a) Phylogenetic distribution of *bioE* genes. Phylogenetic analysis reveals that bioE genes are distributed across bacterial families, including Weeksellaceae, Flavobacteriaceae, Chitinophagaceae, Sphingobacteriaceae, Flexibacteriaceae, Burkholderiaceae, and the obligately parasitic family Chlamydiaceae. b) Ubiquitous occurrence of *bioE* in Chlamydiaceae. Phylogenetic inference across five major *Chlamydia* species within Chlamydiaceae demonstrates universal conservation of the BioE pathway, with the sole exception of *C. trachomatis* c) Evolutionary loss of the biotin synthesis genes in *C. trachomatis*. Linear alignment of genomic loci reveals a specific deletion of the complete biotin biosynthesis gene cluster in *C. trachomatis*. This evolutionary event is hypothesized to correlate with its specialized parasitic lifestyle, possibly reflecting metabolic dependency on host‐derived biotin. d) Genetic context of the biotin biosynthesis gene cluster in *Chlamydia*. Comparative gene cluster analysis shows that most *Chlamydia* species possess a complete biotin biosynthesis operon, including core enzymes for biotin synthesis. However, unlike the *Elizabethkingia*, the *Chlamydia* species lack the transcriptional regulator BioL in the cluster. e) Functional complementation of the *E. coli* BioC‐BioH pathway by BioE In *Elizabethkingia*, the EmBioE mediates cleavage of long‐chain acyl‐group substrates, generating the pimeloyl‐ACP (CoA) precursor required for biotin synthesis. Heterologous expression of CpBioE orthologs demonstrates analogous activity, enabling bypass of the canonical BioC‐BioH pathway in *E. coli*. The experiments were conducted in M9 minimal medium, supplemented with 0.4% glycerol as the carbon source and 0.2% arabinose as the inducer. The experiments were performed in three biological replicates, and one of these is presented as a representative. **Designations**: ACP, acyl carrier protein; EmBioE, *Elizabethkingia mengingoseptica* BioE; CpBioE, *Chlamydia psittaci* BioE; *C. trachomatis*, *Chlamydia trachomatis*. *E. coli*. *Escherichia coli*; ara, L‐arabinose.

The BioE‐mediated biotin biosynthesis pathway was recently identified in nosocomial pathogenic species of the genera *Elizabethkingia* and *Chryseobacterium*, both belonging to the family Weeksellaceae. Using EmBioE as a template, we searched for BioEs in the NCBI database with parameters set at >40% identity and >85% coverage, followed by redundancy removal. Phylogeny revealed that BioE exhibited a broad distribution across several families, including Weeksellaceae, Flavobacteriaceae, Chitinophagaceae, Sphingobacteriaceae, Flectobacillaceae, and Burkholderiaceae, as well as host‐parasitic species within the *Chlamydiaceae* family (**Figure** **1**a). The most common species within the Chlamydiaceae family harbored *bioE*, with the exception of *C. trachomatis* (Figure 1b). Comparative genomic analysis revealed that *C. trachomatis* naturally lacks the biotin biosynthesis gene cluster (Figure 1c), a genetic feature that can serve as a biomarker for identification. By comparing the genetic context of the biotin operon in seven *Chlamydia* species and *E. meningoseptica*, we found that the biotin biosynthesis gene cluster is conserved in *Chlamydia*, but unlike *E. meningoseptica*, the *Chlamydia* genus lacks the transcriptional regulator BioL (Figure 1d). Phylogenetic analysis of genomic traits and host associations across eight common *Chlamydia* species revealed that *Chlamydia trachomatis*, *Chlamydia caviae*, and *Chlamydia muridarum*—with natural hosts being humans or rodents—have lost their biotin biosynthesis gene clusters, while retaining the BioY‐mediated biotin transport pathway (Figure S1a,b, Supporting Information). This loss is likely driven by endogenously high biotin levels in rodent serum and a consistent biotin supply in human hosts.^[^ ^10^ ^]^ These conditions rendered the *de novo* biotin biosynthesis pathway dispensable in these three *Chlamydia* species, resulting in the sole retention of the biotin transport machinery. Furthermore, the *Chlamydia* BioY biotin transporter and mammalian sodium multivitamin transporter (SMVT) function synergistically to facilitate the efficient uptake of biotin and other vitamins into *Chlamydia* cells.^[^ ^40^ ^]^ Notably, all eight *Chlamydia* species consistently retain genes encoding enzymes of the type II fatty acid synthesis (FAS II) pathway (FabD, FabH, FabG, FabZ, and FabI). This pathway not only relies on biotin as a coenzyme but also generates substrates for biotin biosynthesis (Figure S1b, Supporting Information). These findings indicate that the genetic features of the biotin biosynthesis pathway in the *Chlamydia* genus are tightly linked to its host environment. We subsequently cloned CpBioE from *Chlamydia psittaci* (*C. psittaci*), which shares ≈40% sequence identity with EmBioE. Via heterologous complementation with CpBioE, we confirmed that, like EmBioE, CpBioE can bypass the canonical BioC‐BioH pathway in *E. coli* (Figure 1e). In summary, the BioE biotin biosynthesis pathway exhibits both genetic conservation and diversity across multiple taxonomic groups.

### 2.2 Disruption of Repressor BioL Impaired Biotin Biosynthesis and Biofilm Formation in *E. meningoseptica*

**Figure 2:**
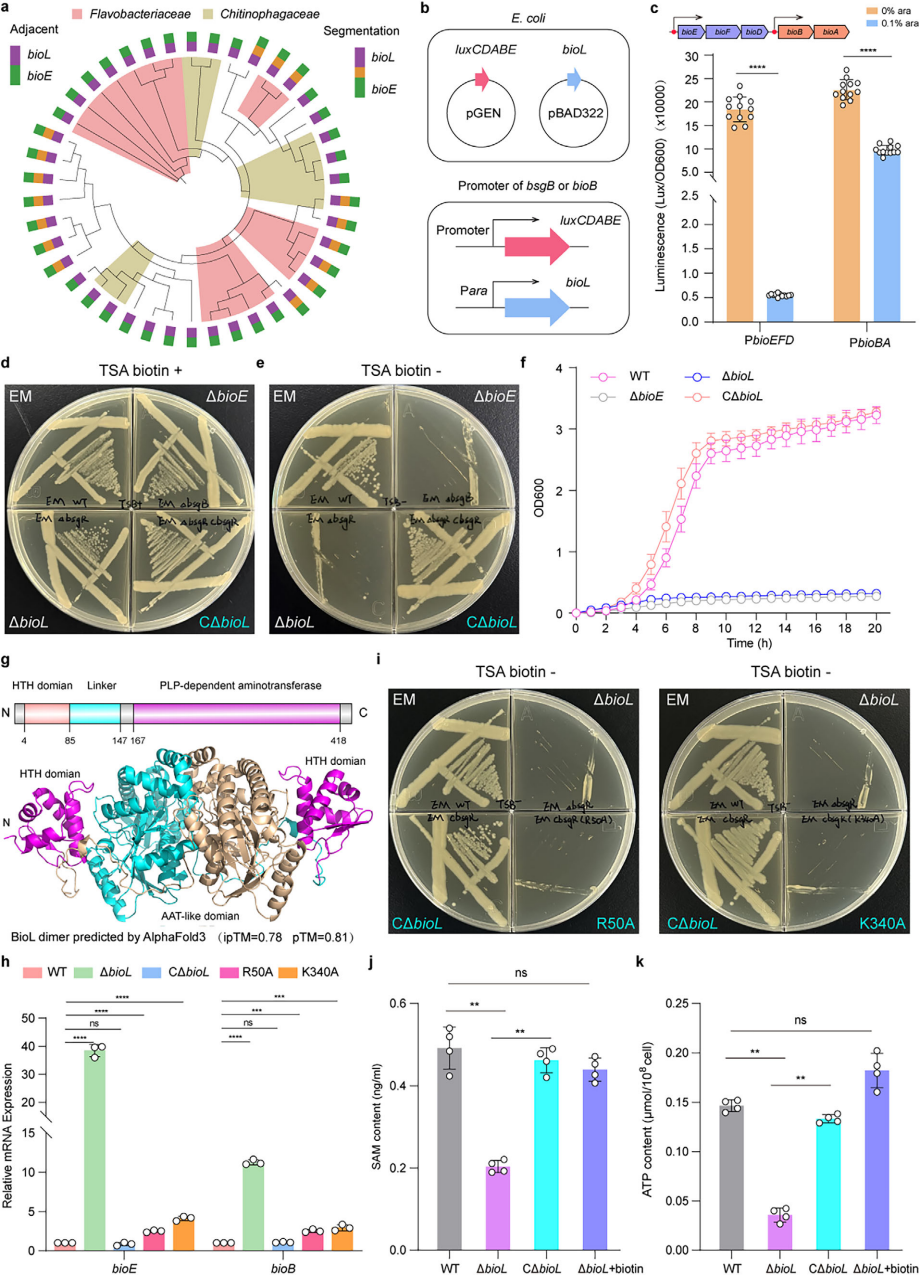
Deletion of repressor BioL induces biotin auxotrophy phenotype in *E. meningoseptica* a) Co‐occurrence analysis of *bioL* and *bioE*. Phylogenetic analysis revealed that the *bioE* gene coexists with *bioL* in the majority of bacterial strains, either adjacent to or dispersed from each other in the genome. b,c) BioL negatively regulates the expression of biotin biosynthesis gene clusters. A lux fluorescent reporter system was constructed to investigate BioL‐mediated regulation of *bioE/F/D* and *bioB/A* in *E. coli* MG1655. The pGEN‐*luxCDABE* plasmid containing the promoter regions of *bioEFD* and *bioBA* was co‐transformed with pBAD322 encoding arabinose‐inducible BioL b). Upon induction with 0.1% arabinose, luminescence signals from *bioE* and *bioB* promoters decreased to 5000 and 10 0000 relative light units (RLU), respectively, compared to 20 0000 RLU in the absence of arabinose c). Data represent mean ± SD from *n* = 4 biological replicates. d,e). The Δ*bioL* strain phenocopies the growth defect of *bioE* mutants under biotin limitation medium. On Tryptic Soy Agar (TSA) medium devoid of biotin, the Δ*bioL* strain exhibited a biotin auxotrophic phenotype identical to that of the Δ*bioE* strain, which was fully rescued by genetic complementation with a functional *bioL* allele. All experiments were performed in three biological replicates, and one of these is presented as a representative. f) Growth curves demonstrate impaired proliferation of Δ*bioL* strain in biotin‐free medium. Cultures of the Δ*bioL* strain failed to grow in biotin‐depleted Tryptic Soy Broth (TSB), confirming its dependence on exogenous biotin for proliferation. Data represent mean ± SD from *n* = 3 biological replicates. g). The dimer structure of BioL predicted by AlphaFold3 The BioL comprises a N‐terminal DNA‐binding domain, a central linker region, and a C‐terminal pyridoxal 5′‐phosphate(PLP)‐dependent aminotransferase domain. EmBioL is a transcription factor belonging to the MocR family and typically exists as a dimer. A high‐confidence dimeric structure of EmBioL was predicted using AlphaFold3. h). RT‐qPCR validates loss of biotin operon repression by BioL mutants. Quantitative RT‐qPCR revealed ≈40‐fold and ≈13‐fold upregulation of *bioE* and *bioB* transcripts, respectively, in the Δ*bioL* strain compared to wild‐type. Corresponding upregulation of ≈4‐fold was observed in both R50A and K340A mutants. Data represent mean ± SD from *n* = 3 independent biological replicates. i). R50 and K340 mutations in BioL confer biotin auxotrophy in *E. meningoseptica*. Residues R50 (a DNA‐binding motif critical for operator interaction) and K340 (the PLP cofactor‐binding site in the aminotransferase domain) were mutated to alanine. Both R50A and K340A mutants exhibited growth arrest on biotin‐free TSA medium, phenocopying the Δ*bioL* strain. The experiment was performed in three biological replicates, and one of these is presented as a representative. j). BioL deletion reduces intracellular SAM levels in *E. meningoseptica* ELISA quantification of intracellular SAM showed a significant decrease from ≈0.5 ng mL^−1^ in wild‐type to ≈0.2 ng mL^−1^ in the Δ*bioL* strain. Genetic complementation with *bioL* or exogenous 2 mm biotin supplementation fully restored SAM levels to wild‐type. Data represent mean ± SD from *n* = 4 biological replicates k). Loss of BioL impairs intracellular ATP homeostasis of *E. meningoseptica*. ATP Content Determination revealed a reduction from ≈0.15 µmol 10^−8^ cells in wild‐type to ≈0.04 µmol 10^−8^ cells in the Δ*bioL* strain. Restoration of ATP levels was achieved via *bioL* complementation or 2 mm biotin supplementation. Data represent mean ± SD from *n* = 4 biological replicates. All the statistical significance was determined by unpaired two‐tailed Student's *t*‐test (^**^ *p* < 0.01, ^***^ *p* < 0.001, ^****^ *p* < 0.0001).

In *Elizabethkingia*, BioL negatively regulates the biotin biosynthesis pathway. Gene co‐occurrence analysis showed that BioL is prevalent in most species harboring the BioE pathway, either adjacent to or spatially separated from the “*bio* operon” (**Figure** **2**a). Using a fluorescence reporter system, co‐transformation of BioL with reporters containing *bioEFD* or *bioBA* promoter regions revealed significant fluorescence reduction upon 0.1% arabinose induction, indicating BioL represses *bioEFD* and *bioBA* expression (Figure 2b,c). Markerless *bioL* deletion in *E. meningoseptica* generated a Δ*bioL* strain with a biotin auxotrophic phenotype identical to that of the Δ*bioE* strain cultured on biotin‐free Trypticase Soy Agar (TSA) medium (Figure 2d,e). A similar phenotype was observed in *C. indologenes*, where deletion of Ci*bioL* resulted in biotin auxotrophy (Figure S2a,b, Supporting Information). To exclude the possibility that EmBioL functions as an accessory gene (analogous to BsaP) involved in biotin biosynthesis, a heterologous complementation system was established. Specifically, BioF from *E. meningoseptica* (EmBioF) and BioF from *C. psittaci* (CpBioF) were heterologously expressed in *E. coli* Δ*bioF* and Δ*bioH*/*F* strains, respectively. CpBioF successfully complemented the biotin auxotrophic phenotype of *E. coli* Δ*bioF*, whereas EmBioF failed to do so. However, both EmBioF and CpBioF were able to complement the biotin auxotrophy of *E. coli* Δ*bioH*/*F* when BioW was co‐expressed and exogenous pimelic acid was supplemented^[^ ^32^ ^]^(Figure S2c,d, Supporting Information). This indicates that EmBioF could only recognize pimeloyl‐CoA as a substrate in vivo,^[^ ^37^ ^]^ and confirming that EmBioL is not an accessory protein for BioF. A growth curve further confirmed that the Δ*bioL* strain could not grow in biotin‐depleted TSB (Figure 2f), prompting an investigation into why deleting a negative regulator caused biotin auxotrophy.

BioL comprises three domains: an N‐terminal DNA‐binding domain, a central linker, and a C‐terminal pyridoxal phosphate (PLP‐)‐dependent aminotransferase domain (AAT‐like domain). AlphaFold3 predicted a high‐confidence dimeric structure for BioL (ipTM = 0.78, pTM = 0.81) (Figure 2g). The expression and purification of EmBioL, coupled with EMSA and RT‐qPCR analyses, further confirmed that the regulator BioL binds directly to the promoter regions of the biotin biosynthesis gene clusters (*bioEFD* and *bioBA*) and negatively regulates their expression (Figure 2h; Figure S2e–h, Supporting Information). Sequence alignment of EmBioL with the well‐characterized regulators *Bacillus subtilis* GabR (BsGabR) and *Bacillus clausii* PdxR (BcPdxR) revealed that, despite sharing < 20% sequence identity, all three proteins belong to the MocR family of transcription factors and contain multiple conserved residues.^[^ ^41^, ^42^ ^]^ These include R50 within the helix‐turn‐helix (HTH) motif (which mediates DNA interactions) and K340 in the AAT‐like domain (predicted to covalently bind to PLP).^[^ ^37^ ^]^ Molecular docking of PLP into the AAT‐like domain of EmBioL, followed by structural comparison with known GabR and PdxR structures, identified numerous spatially conserved residues involved in PLP interactions (Figure S3a–d, Supporting Information). Site‐directed mutations of R50A or K340A phenocopied the Δ*bioL* auxotrophic phenotype on biotin‐depleted TSA (Figure 2i). RT‐qPCR results showed significant upregulation of *bioEFD* and *bioEA* in R50A, K340A, and Δ*bioL* strains; Complementation restored wild‐type expression levels (Figure 2i; Figure S2e, Supporting Information).

Given the high metabolic cost of biotin synthesis in terms of SAM and ATP consumption,^[^ ^43^ ^]^ we hypothesized that *bioL* deletion would lead to excessive depletion of these molecules. We quantified SAM and ATP levels in wild‐type, Δ*bioL*, CΔ*bioL*, and Δ*bioL* strains supplemented with 2 mm biotin. In the Δ*bioL* strain, SAM and ATP levels were significantly depleted to ≈0.2 ng mL^−1^ and ≈0.04 µm/10^8^ cells, respectively. Both CΔ*bioL* and exogenous 1 mmm biotin supplementation restored SAM and ATP to wild‐type levels (Figure 2j,k). Subsequently, cluster analysis of *E. meningoseptica* transcriptome profiles under biotin‐supplemented and biotin‐depleted conditions revealed significant upregulation of ATP biosynthesis pathway genes in biotin‐depleted cultures (Figure S4a, Supporting Information). Biofilm formation assays showed that both Δ*bioL* and Δ*bioE* strains exhibited markedly reduced biofilm production compared to wild‐type and complemented strains (Figure S4b, Supporting Information). Confocal microscopy further confirmed diminished biofilm formation in the Δ*bioL* strain (Figure S4c, Supporting Information). These findings demonstrate that *bioL* mutations disrupt metabolic homeostasis in *E. meningoseptica*, affecting both biotin biosynthesis and biofilm formation.

### 2.3 Structural Conservation and Specificity of CpBioE and EmBioE

**Figure 3:**
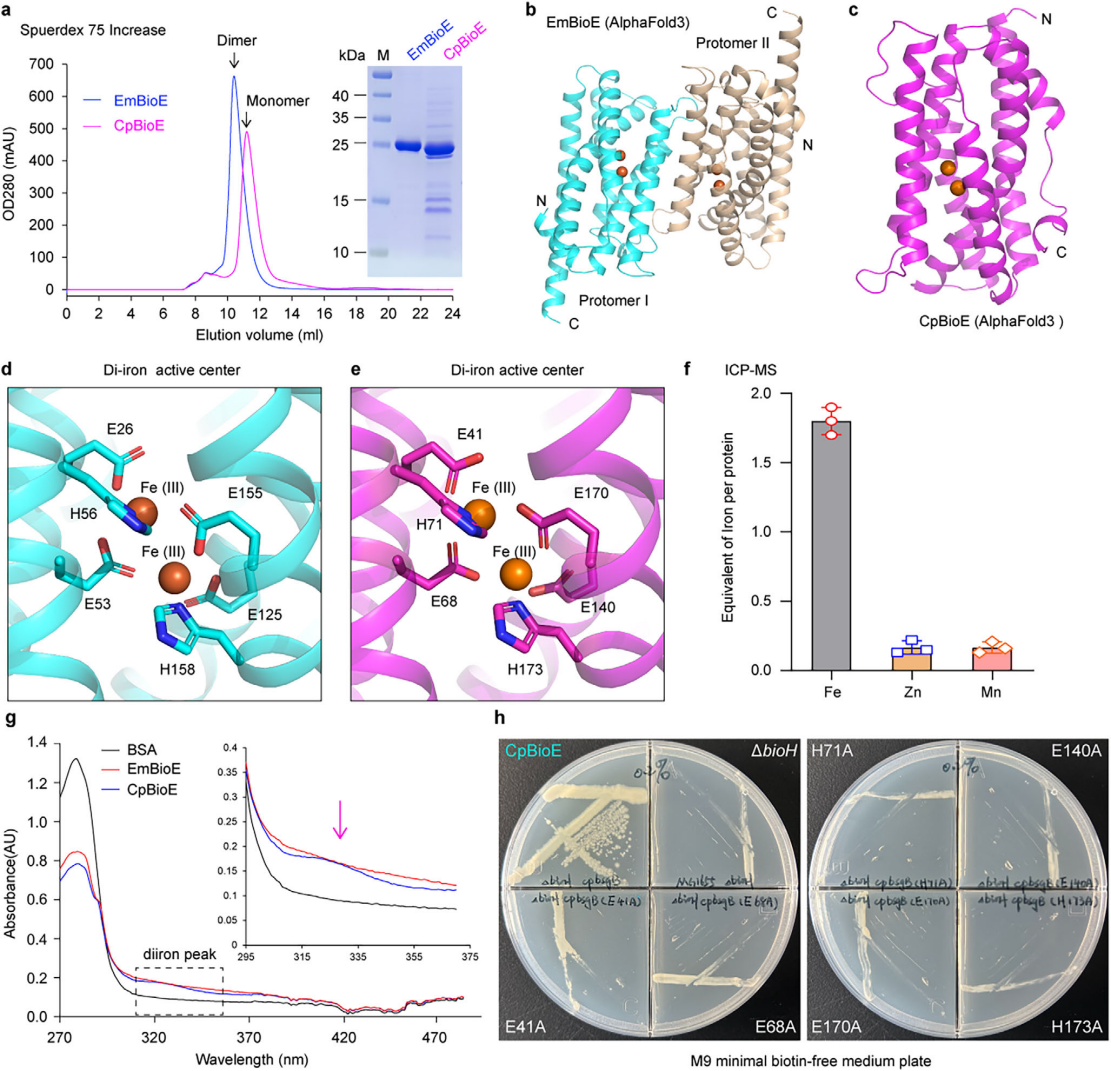
The structural characterization of CpBioE and EmBioE a). Size‐exclusion chromatography of EmBioE and CpBioE. Size‐exclusion chromatography results revealed that, based on the retention volume on a Superdex 75 Increase column, EmBioE elutes as a dimer, whereas CpBioE exhibits a monomeric configuration. b,c). AlphaFold3‐predicted structural features of EmBioE and CpBioE d). EmBioE contains a conserved di‐iron active center organized by E26, E53, H56, E125, E155, and H158 e). CpBioE harbors a diiron center coordinated by six residues (E41, E68, H71, E140, E170, and H173), forming the catalytic core f). ICP‐MS quantification of iron content in CpBioE Inductively coupled plasma mass spectrometry (ICP‐MS) confirmed the presence of ≈2 iron atoms per CpBioE monomer, with negligible Mn or Zn content. g). UV‐spectroscopy reveals that the BioE protein exhibits characteristic peaks for the diiron center near 335 nm. h). Mutagenesis of active site residues abolishes CpBioE function. Site‐directed mutagenesis of CpBioE at its diiron‐coordinating residues (E41A, E68A, H71A, E140A, E170A, and H173A) abolished its ability to complement the biotin auxotrophy of the *E. coli* Δ*bioH* strain when cultured on M9 minimal medium. Three independent experiments were carried out, and one representative experiment is shown.

Compared to *E. meningoseptica*, *Chlamydia* species naturally lack the regulator BioL, a genotype that warrants further exploration (Figure 1b). We hypothesized that functional divergence exists between EmBioE and CpBioE. Protein purification and size‐exclusion chromatography confirmed EmBioE and CpBioE existed as dimers and monomers, respectively (**Figure** **3**a). High‐confidence AlphaFold3 modeling revealed that EmBioE exhibited a rotationally symmetrical dimeric interface, whereas CpBioE remained monomeric (Figure 3b,c; Figure S5, Supporting Information). Structural analysis showed that both enzymes possessed a diiron active site (Figure 3d,e), with the conserved residues E41, E68, H71, E140, E170, and H173 coordinating two Fe atoms (Figure 3e). Inductively coupled plasma mass spectrometry (ICP‐MS) confirmed ≈2 Fe atoms per CpBioE molecule, with negligible Zn and Mn contents (Figure 3f). UV‐spectroscopy also revealed a characteristic diiron absorption peak near 335 nm (Figure 3g), putatively confirming their classification as diiron oxygenases. Site‐directed mutagenesis of the six conserved residues (E41, E68, H71, E140, E170, and H71) to alanine abrogated the ability of CpBioE to rescue *E. coli* Δ*bioH* growth on biotin‐limited M9 minimal medium, as shown by plate streaking (Figure 3h).

BioE belongs to a family of ferritin‐like oxygenases that catalyze the conversion of long‐chain acyl substrates into the biotin precursor pimeloyl moiety. Structural analysis of EmBioE revealed a conserved binding pocket for long‐chain acyl groups, that was mirrored by the homologous hydrophobic cavity in CpBioE, as indicated by vacuum surface charge density mapping (Figure S6a,b, Supporting Information). Molecular docking simulations revealed that stearic acid was accommodated within this hydrophobic channel, with fatty acid entering the active site of BioE from the omega end (Figure S4c, Supporting Information). This interaction was stabilized by the W33, L37, L40, I48, L105, V135, I139, L146, Y147, Y150, and L205 residues (Figure S6d, Supporting Information). Substitution of these residues with alanine abolished the ability of CpBioE to complement the growth of *E. coli* Δ*bioH* on biotin‐limited medium (Figure S6e, Supporting Information), confirming the critical role of this hydrophobic interface in substrate recognition. Taken together, these data indicate that CpBioE and EmBioE exhibit both structural conservation and divergence.

### 2.4 CpBioE Exhibits Specificity for Long‐Chain Acyl‐ACPs Over Acyl‐CoAs

**Figure 4:**
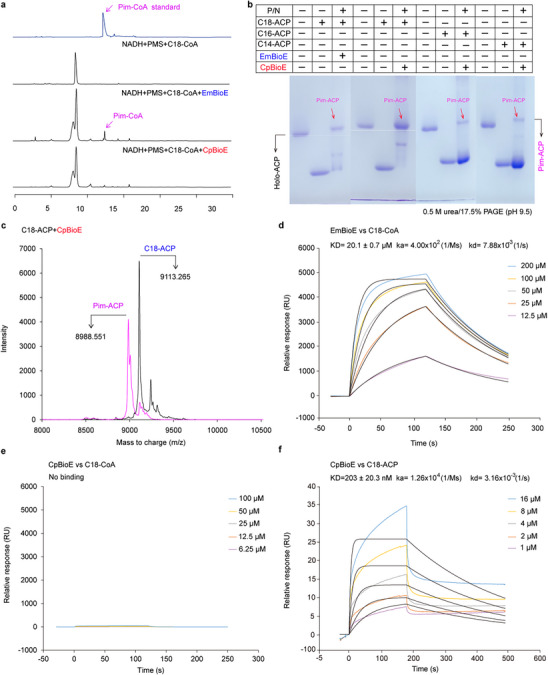
Substrate specificity differences between EmBioE and CpBioE a). HPLC confirms CpBioE cannot catalyze stearoyl‐CoA. HPLC analysis showed that EmBioE could catalyze stearoyl‐CoA(C18‐CoA) to generate pimeloyl‐CoA(C7‐CoA), whereas CpBioE failed to produce detectable C7‐CoA from C18‐CoA. Commercially available Pimeloyl‐CoA (MCE, CAS No.:18907‐20‐5) was used as a reference standard. b). Urea‐PAGE reveals CpBioE recognizes long‐chain acyl‐ACP substrates. Urea‐PAGE demonstrated that both EmBioE and CpBioE catalyze long‐chain acyl‐ACP (C14‐ACP, C16‐ACP, and C18‐ACP) to produce pimeloyl‐ACP (Pim‐ACP), with CpBioE exhibiting the highest catalytic efficiency toward C18‐ACP. c). MALDI‐TOF identifies pimeloyl‐ACP as the product of CpBioE‐catalyzed stearoyl‐ACP. MALDI‐TOF mass spectrometry of CpBioE reaction products revealed a dominant peak at m/z 8988.551, which corresponds to the molecular weight of Pim‐ACP. In comparison, stearoyl‐ACP exhibits an m/z value of 9113.265. d). SPR‐based kinetic analysis was performed to characterize the interaction between EmBioE and stearoyl‐CoA. SPR analysis revealed that EmBioE binds C18‐CoA with a dissociation constant (KD) of ≈20.1 µM e). SPR kinetic analysis was conducted to evaluate the interaction between CpBioE and stearoyl‐CoA (C18‐CoA). No specific binding signal was detected, confirming the absence of interaction between CpBioE and C18‐CoA f). SPR‐based kinetic measurements were performed to determine the binding between CpBioE and C18‐ACP, with a KD of ≈203 nm, demonstrating recognition of the acyl‐ACP substrate over CoA derivatives. The SPR experiments were performed three independent times, and a representative SPR profile is presented herein. For the SPR data, kinetic analysis was employed. Given that the substrate was not saturated, the black curve represents the fitted curve, while the colored curves denote the raw data. The concentrations of the substrate are indicated in the figure. **Designations**: HPLC, high performance liquid chromatography; P/N, PMS, (phenazine methosulfate) and NADH; C18‐CoA, stearoyl‐CoA; C14‐ACP, myristoyl‐ACP; C16‐ACP, palmitoyl‐ACP; C18‐ACP, stearoyl‐ACP; Pim‐CoA, pimeloyl‐CoA; Pim‐ACP, pimeloyl‐ACP; MALDI‐TOF, matrix‐assisted laser desorption ionization time‐of‐flight mass spectrometry.

Notably, EmBioE demonstrated dual catalytic activity toward both long‐chain acyl‐ACPs and acyl‐CoAs, generating pimeloyl‐ACP or pimeloyl‐CoA in the PMS/NADH chemical redox partners (**Figure** **4**a,b). In contrast, High Performance Liquid Chromatography (HPLC) analysis revealed that CpBioE did not generate pimeloyl‐CoA from stearoyl‐CoA(C18‐CoA), unlike EmBioE (Figure 4a). Acyl‐ACPs of varying chain lengths were generated using *Vibrio harveyi* acyl‐ACP synthetase (VhAasS) and *E. coli* holo‐ACP (Figure S7a,b, Supporting Information). Urea‐PAGE analysis, with EmBioE‐cleaved C18‐ACP and BioH‐cleaved E‐C7‐ACP as controls, showed CpBioE processed myristoyl‐ACP(C14‐ACP), palmitoyl‐ACP(C16‐ACP), and stearoyl‐ACP(C18‐ACP) into pimeloyl‐ACP, with optimal activity toward C18‐ACP (Figure 4b, Figure S7d, Supporting Information). MALDI‐TOF mass spectrometry confirmed that the product exhibited an m/z value of 8988.551, which matched the calculated mass of pimeloyl‐ACP (Pim‐ACP) (Figure 4c). Before the reaction, the m/z value of C18‐ACP was 9113.265. We also determined the m/z values of E‐C7‐ACP before and after cleavage by BioH as 9015.449 and 8988.756, respectively (Figure 4c; Figure S7c, Supporting Information). The mass of product was consistent with that of the CpBioE‐catalyzed cleavage of C18‐ACP, further confirming that the enzymatic product of CpBioE was Pim‐ACP. Surface plasmon resonance (SPR) revealed EmBioE bound C18‐CoA with a KD of ≈20.1 µM, whereas CpBioE exhibited no significant binding to C18‐CoA (Figure 4d,e). Conversely, CpBioE binds to C18‐ACP with a KD of ≈203 nM (Figure 4f). Furthermore, validation by microscale thermophoresis (MST) confirmed the affinity of EmBioE for C18‐CoA and CpBioE for C18‐ACP, which was consistent with their respective substrate specificities (Figure S8, Supporting Information). Overall, these findings establish the exclusive recognition of long‐chain acyl‐ACPs over acyl‐CoAs by CpBioE.

Molecular docking experiments predicted that EmBioE forms a complex with C18‐CoA (Figure S9a, Supporting Information). Detailed analysis of the CoA‐binding interface identified a positively charged region in EmBioE comprising F24, R31, K32, K46, R87, Q88, and H91, which interacted with the negatively charged CoA moiety (Figure S9b, Supporting Information). Structural comparison suggested that the corresponding region in CpBioE contains positively charged residues K46, K47, K102, acidic residue D106, and hydrophobic residues F39 and Y103 (Figure S9c, Supporting Information). For further analysis, EcACP was extracted from the FabB‐EcACP complex (PDB: 7SZ9)^[^ ^44^ ^]^ and docked with CpBioE; this result also revealed that the negatively charged helix 2 of EcACP primarily interacts with the positively charged region of CpBioE (Figure S9d, Supporting Information). Site‐directed mutagenesis of these six residues in CpBioE demonstrated that single‐site mutations abolished its ability to rescue *E. coli* Δ*bioH* growth on biotin‐depleted medium plates (Figure S9e, Supporting Information). These results may reveal that residue differences at this interface between EmBioE and CpBioE underlie their divergent substrate specificities.

Ferritin‐like diiron oxygenases require oxygen and redox systems for their catalytic reactions.^[^ ^45^ ^]^ Consistent with this, our urea‐PAGE analysis revealed that CpBioE required the presence of PMS and NADH (as electron donors) to fully convert the substrate C18‐ACP (Figure S10a, Supporting Information). We further confirmed via GC‐MS that, in addition to Pim‐ACP, undecanoic acid was present as a product of CpBioE‐catalyzed C18‐ACP cleavage (Figure S10b, Supporting Information). Under anaerobic culture conditions: i) *Elizabethkingia* failed to grow on TSA with or without biotin; ii) *E. coli* Δ*bioH* strains complemented with EmBioE or CpBioE failed to restore the biotin auxotrophic phenotype under anaerobic conditions, whereas they successfully restored growth under aerobic conditions (Figure 1e; Figure S10c,d, Supporting Information). These results indicate that BioE requires oxygen for its enzymatic activity. Combining the above biochemical and genetic evidence, and referencing the known catalytic mechanism of stearoyl‐ACP desaturase and BioI(P450),^[^ ^46^, ^47^ ^]^ we hypothesized that CpBioE mediates the synthesis of the biotin precursor Pim‐ACP under O_2_ and NADH‐dependent conditions via a series of reactions: desaturation, sequential hydroxylation, oxidative cleavage, and subsequent oxidation (Figure S10e, Supporting Information). However, the exact catalytic mechanism of this reaction requires further investigation.

### 2.5 Fitness Cost of EmBioE Overexpression in Bacterial Cells

**Figure 5:**
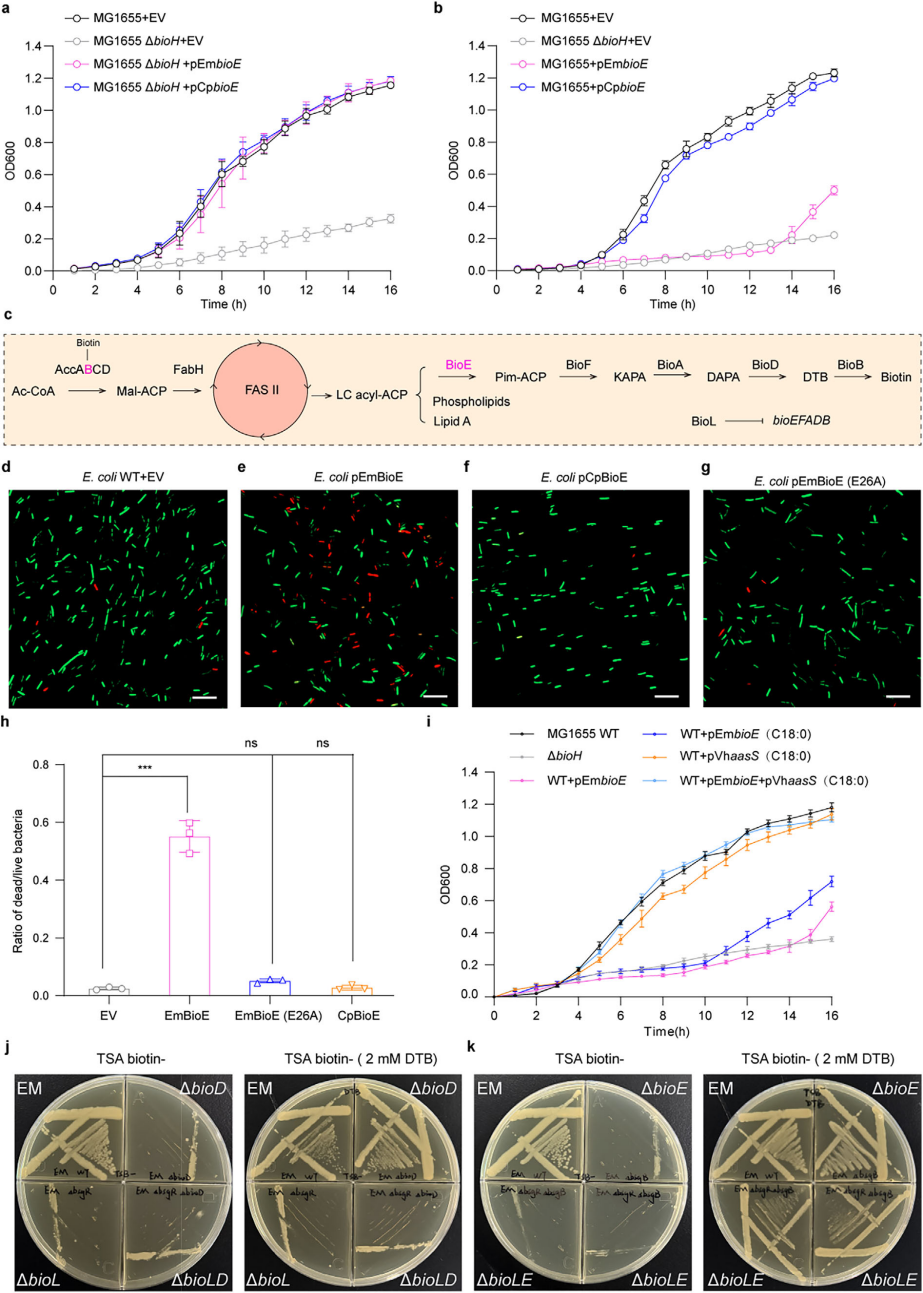
Effects of *bioE* overexpression on bacterial cell viability a). BioE restores growth of *E. coli* Δ*bioH* in biotin‐free M9 minimal medium. Growth curve analysis showed that EmBioE and CpBioE expression induced by 0.2% arabinose rescued the biotin auxotrophic phenotype of *E. coli* MG1655 Δ*bioH* when cultured in biotin‐depleted M9 medium. b). Overexpression of EmBioE induces growth arrest in wild‐type *E. coli*, whereas CpBioE overexpression has no such effect. Under induction with 0.2% arabinose, wild‐type *E. coli* MG1655 harboring plasmid pBAD‐EmBioE exhibited a growth arrest phenotype due to EmBioE overexpression. In contrast, wild‐type *E. coli* MG1655 harboring plasmid pBAD‐CpBioE did not display this phenotype when CpBioE was overexpressed under the same induction conditions. All growth curves (a and b) were presented as the means ± SD, with data derived from three independent experiments. c). Schematic diagram of BioE‐mediated lipid metabolism in bacteria d–g). Confocal microscopy reveals increased cell death in EmBioE‐overexpressing *E. coli* MG1655. Confocal laser scanning microscopy images of strains carrying the EmBioE derivatives and CpBioE stained with LIVE/DEAD kit (*n* = 3). Live and dead cells presented green and red color, respectively. Scale bar is 5 µm. h). Ratio of dead to live bacterial cell obtained from CLSM z‐stack images. The data is given as the mean ± SD (*n* = 3), and assessed by unpaired two‐tailed Student's *t*‐test. (^***^ *p* < 0.001; ns, not significant). i). Exogenous long‐chain fatty acid supplementation alleviates EmBioE‐induced cellular damage. Plasmid pET28a‐Vh*aasS* was transformed into wild‐type *E. coli* MG1655 and *E. coli* MG1655 harboring plasmid pBAD‐EmBioE, respectively. In the culture system, 0.2% arabinose was added to induce EmBioE expression. In *E. coli* MG1655+pEmBioE supplemented with 2 mm stearic acid, no significant recovery of its growth retardation phenotype was observed. For *E. coli* MG1655+pVhAasS, supplementation with 2 mm stearic acid had no effect on bacterial growth. In contrast, when stearic acid was supplemented to *E. Coli*. MG1655+pEmBioE+pVhAasS, the bacterial growth retardation phenotype was restored. These results indicate that the host bacterial growth arrest caused by EmBioE overexpression is likely attributed to the depletion of long‐chain lipids. The growth curves are presented as the means ± SD, with data derived from three independent experiments. j,k). EmBioE overexpression disrupts biotin metabolism in *Elizabethkingia*. In *Elizabethkingia*, deletion of *bioL* and/or *bioD* caused biotin auxotrophy. Supplementation with dethiobiotin (DTB, a BioD reaction product) rescued the phenotype of Δ*bioD* mutants but failed to restore growth in Δ*bioL* or Δ*bioL*/Δ*bioD* double mutants j). DTB also rescued the biotin auxotrophy of Δ*bioE* mutants, whereas the Δ*bioL*/Δ*bioE* double mutant remained DTB‐responsive k). All experiments included three independent replicates, with one replicate selected to be displayed as a representative. **Designations**: EV, Empty vector.

Complementation of *E. coli* Δ*bioH* with EmBioE or CpBioE restored growth on biotin‐free M9 medium (**Figure** **5**a). Notably, when heterologously expressed in wild‐type *E. coli* with 0.02%, 0.05%, and 0.2% arabinose induction, only EmBioE overexpression caused growth retardation (Figure 5b; Figure S11a, Supporting Information). A similar phenotype is also evident in the BioC‐BioH pathway of *E. coli*, where high expression of BioC blocks fatty acid synthesis.^[^ ^20^ ^]^ Additionally, we examined the enzymes involved in the late‐stage biotin biosynthetic pathway (BioF, BioA, BioD, and BioB) in *Elizabethkingia*. When these four proteins were overexpressed in *E. coli* MG1655, only BioB exerted a weak effect on host growth, and EmBioE exerted the most prominent growth retardation effect (Figure 5b; Figure S11b, Supporting Information). This phenotype is intriguing, as EmBioE rescues Δ*bioH* growth while impairing wild‐type viability under biotin‐free M9 medium conditions. In bacteria, fatty acid synthesis relies on biotinylated AccB to convert acetyl‐CoA into long‐chain fatty acids via the FAS II pathway, which is essential for membrane phospholipid, lipopolysaccharide, and biotin biosynthesis (Figure 5c). Thus, EmBioE overexpression likely disrupted lipid metabolic homeostasis. Live/Dead staining (green, viable cells; red, dead cells) combined with confocal microscopy revealed a significant increase in dead cells in EmBioE‐overexpressing cultures compared to those in the empty vector controls (Figure 5d,e). In contrast, CpBioE overexpression had no detectable effect (Figure 5f). The expression of the catalytically inactive EmBioE(E26A) mutant did not impose a fitness cost (Figure 5g). Quantitative analysis showed EmBioE overexpression induced ≈37% cell death, which was a significant increase compared to CpBioE or EmBioE(E26A) (Figure 5h).

In the *E. coli* MG1655 background, we reconstituted the Vh*aasS* gene, which enables uptake of exogenous fatty acids into the *E. coli* fatty acid synthesis pathway.^[^ ^48^ ^]^ Plasmids pET28a‐Vh*aasS* and pBAD322‐Em*bioE* were co‐transformed into *E. coli* MG1655. L‐arabinose was added at a final concentration of 0.2% to induce EmBioE expression, whereas VhAasS relied on basal‐level expression.^[^ ^21^ ^]^ Compared with the conditions where EmBioE was expressed alone or EmBioE was expressed with the addition of stearic acid, the combination of the strain co‐expressing VhAasS and EmBioE supplemented with 0.2 mm stearic acid significantly restored the growth of the host strain (Figure 5i). GC‐MS analysis revealed that EmBioE overexpression depleted long‐chain fatty acids (C14:0, C14:1, C16:0, C16:1, C18:0, and C18:1) compared to empty vector controls (Figure S11c,d, Supporting Information), indicating that EmBioE disrupts lipid homeostasis and impedes bacterial growth. To test whether EmBioE overexpression underlies the biotin auxotrophy of *Elizabethkingia* Δ*bioL*, we performed epistasis analysis. Deletion of *bioD*, a late‐stage biotin biosynthesis gene, was rescued by 2 mm DTB on biotin‐limited TSA medium, whereas Δ*bioL* and Δ*bioL*Δ*bioD* mutants remained auxotrophic (Figure 5j). In contrast, Δ*bioE* and Δ*bioL*Δ*bioE* mutants were DTB‐responsive (Figure 5k), though the double mutant showed reduced growth relative to the Δ*bioE* strain. This observation is likely due to overexpression of the *bioF*/*bioA*/*bioD*/*bioB* genes, which disrupts bacterial metabolism—specifically, ATP and SAM homeostasis (Figure 2j,k). Collectively, these results confirm that BioL‐mediated repression of EmBioE or “*bio* operon” is critical for maintaining lipid homeostasis in *Elizabethkingia*.

### 2.6 Virtual Screening of Lead Inhibitors Against BioE

**Figure 6:**
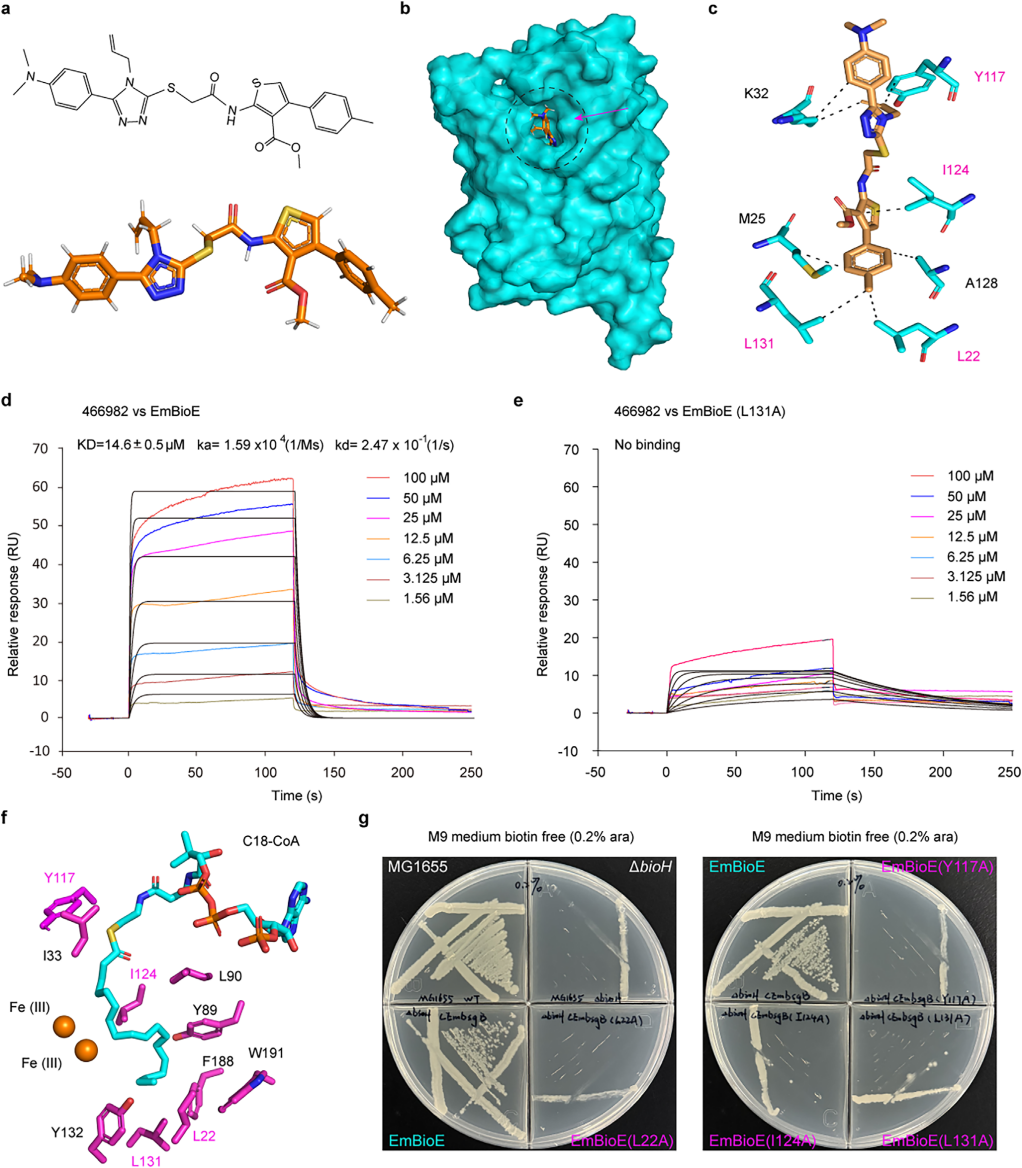
The interactions between BioE and inhibitor 466982 identified via virtual screening a). Chemical structure of compound 466982 The chemical formula of small molecule 466982 is presented, highlighting its aromatic and aliphatic moieties. b). Molecular docking reveals 466982 occupies the substrate‐binding pocket of BioE In silico docking analysis predicted that compound 466982 binds within the conserved substrate‐binding cavity of BioE, overlapping with the acyl‐CoA binding site. c). Structural illustration for the interaction between BioE and 466982. Six amino acid residues (L22, M25, K32, Y117, I124, and L131) form hydrophobic interactions with compound 466982, as predicted by molecular dynamics simulations. d). SPR kinetic analysis revealed direct binding between 466982 and BioE Sensorgrams were fitted to a 1:1 Langmuir binding model, yielding an association rate constant ka of ≈1.59 × 10^4^ M^−1^s^−1^, a dissociation rate constant kd of ≈1.16 × 10^−4 ^s^−1^, and a corresponding equilibrium dissociation constant (KD = kd/ka) of ≈14.6 µm. e) The L131 mutation in BioE abolishes binding affinity for compound 466982. The L131A mutation in BioE caused a significant reduction in binding affinity for compound 466982, such that no detectable KD was observed via SPR f). Hydrophobic interactions between BioE and the acyl chain of C18‐CoA BioE's binding pocket engages the hydrophobic acyl chain of C18‐CoA through residues L22, I33, Y89, L90, Y117, I124, L131, F188, and W191, forming a hydrophobic tunnel. g). Mutating residues in BioE that interact with compound 466982 abolishes its enzymatic function. Substitution of residues L22, Y117, I124, and L131 (which interact with both 466982 and C18‐CoA) completely abrogated the ability of BioE to complement the biotin auxotrophy of *E. coli* Δ*bioH*. Three independent replicates were conducted for each experiment, and one of these was chosen for presentation as a representative. A representative surface plasmon resonance (SPR) affinity profile from three independent experiments (*n* = 3) is shown.

*Elizabethkingia*, a multidrug‐resistant emerging pathogen, requires novel antimicrobial therapies.^[^ ^49^ ^]^ The enzymes in biotin biosynthesis are attractive antimicrobial targets. Given the conservation of BioE in pathogenic *Elizabethkingia*, *Chryseobacterium*, and *Chlamydia*, screening for small‐molecule BioE inhibitors offers therapeutic promise for these pathogens. Virtual screening based on a structural model of BioE was conducted using the DrugFlow platform (developed by Carbon SiliconAI). Using KarmaDock and CarsiDock docking algorithms, results were ranked by the AI‐based RTMScore rescoring algorithm, followed by molecular dynamics simulations with MM/GBSA calculations, yielding a prioritized list of top 10 compounds (Table S3, Supporting Information). Subsequently, we synthesized the aforementioned 10 compounds via TargetMol. Subsequent SPR screening identified compound 466982 as the lead inhibitor, which exhibits both potent binding affinity and optimal solubility. Its chemical structure features two hydrophobic benzene rings (**Figure** **6**a). Molecular docking revealed that 466982 occupies the hydrophobic substrate pocket of BioE, forming critical hydrophobic interactions with residues L22, M25, K32, Y117, I124, A128, and L131 (Figure 6b,c). SPR analysis revealed that compound 466982 binds to EmBioE with a dissociation constant (KD) of ≈14.6 µm, whereas mutation of the residue L131 abolished this interaction (Figure 6d,e). Molecular docking simulations of BioE with C18‐CoA identified L22, I124, Y117, and L131 as key residues mediating acyl‐tail interactions, which are also critical for 466982 binding (Figure 6c,f). These four residues are evolutionarily conserved across known BioE homologs (Figure S12, Support Information). The site‐directed mutagenesis of L22A, I124A, Y117A, and L131A completely abrogated the enzymatic activity of BioE (Figure 6g), confirming that 466982 is a potential inhibitor of the substrate‐binding pocket of BioE.

### 2.7 Potential Antimicrobial Activity of Compound 466982

**Figure 7:**
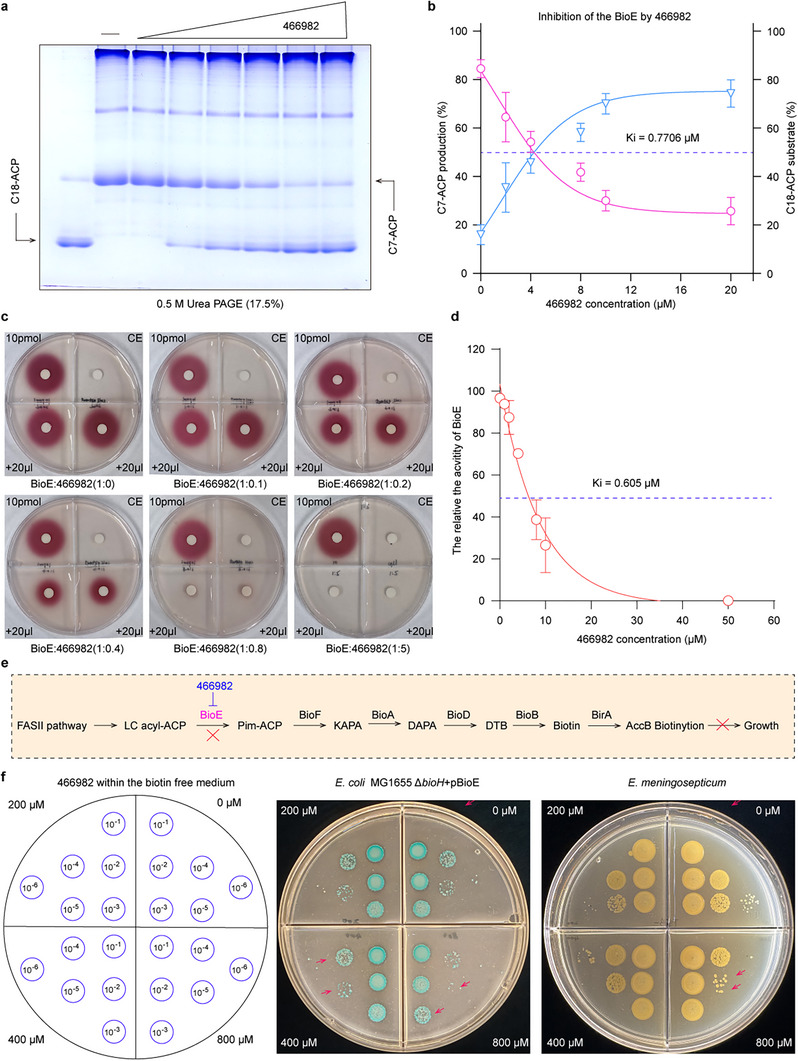
Inhibition of EmBioE enzymatic activity by compound 466982 a). Compound 466982 dose‐dependently inhibits EmBioE‐mediated cleavage of C18‐CoA in vitro C18‐ACP and C7‐ACP were separated by 0.5 m urea/17.5% PAGE (pH 9.5). In vitro enzyme assays showed decreased production of C7‐ACP with increasing 466982 concentrations (2, 4, 8, 10, 20 µm), as visualized by urea‐PAGE. Experiments were performed in triplicate (*n* = 3). b). Relative quantification of the inhibition constant (Ki) for 466982 against EmBioE Steady‐state kinetic analysis yielded a Ki value of 0.7706 µm for 466982‐mediated inhibition of BioE. The left *y*‐axis represents the decrease in the product pimeloyl‐ACP as the concentration of the inhibitor 466982; the right y‐axis represents the decrease in the consumption of the substrate C18‐ACP with increasing concentration of this inhibitor. Data were fitted using nonlinear regression with GraphPad Prism 8.0, and error bars represent standard deviation from three independent experiments. c). In vitro biotin reconstitution assay demonstrates reduced EmBioE‐dependent biotin synthesis by 466982. Treatment with 466982 significantly decreased biotin production in a reconstituted biosynthetic reaction initiated by BioE. A representative image from three independent bioassays is shown. d). Determination of the Ki for 466982 based on cell viability. The Ki value of 466982 was determined via cell viability assays in *E. coli* Δ*bioH* strains. Three independent experiments were conducted to generate dose‐response curves, with data analyzed by nonlinear regression using GraphPad Prism 8.0. Each data point represents the mean ± SD of triplicate measurements (*n* = 3), yielding a calculated Ki of ≈0.605 µm. e). Schematic model of BioE inhibition disrupting bacterial biotin biosynthesis. A mechanistic diagram illustrates how 466982 binding to BioE impedes the catalytic cleavage of acyl‐ACP substrates, thereby blocking biotin precursor formation in the biosynthetic pathway. f). Dose‐dependent growth inhibition of EmBioE‐expressing strains by 466982 Biotin auxotrophy assays were performed in *E. coli* Δ*bioH* strains harboring a plasmid encoding BioE, using biotin‐free M9 minimal medium supplemented with increasing concentrations of 466982 (0, 200, 400, 800 µm). Log‐phase cultures were serially diluted 10‐fold and spotted onto X‐gal‐containing M9 agar plates. Blue colonies, indicative of *E. coli* Δ*bioH* strains heterologous expressing BioE, were visualized via X‐gal cleavage. Viable colonies were marked with arrows. Yellow colonies represent wild‐type *E. meningoseptica* strains naturally expressing BioE. Representative data from three independent experiments is shown. **Designations**: CE, Cell extract.

BioE catalyzes the conversion of long‐chain acyl groups to the biotin precursor pimeloyl‐ACP (Figure 4b). With a KD of 14.6 µm for EmBioE, nearly five‐fold lower than the KD of 20.1 µm compared to its natural substrate C18‐CoA (Figure 4d), suggesting compound 466982 was hypothesized to act as an inhibitor. Urea‐PAGE analysis of C18‐ACP cleavage, with fixed substrate (2 µg) and enzyme (15 µm) concentrations, revealed dose‐dependent inhibition by 466982 (0–20 µm), with a calculated inhibition constant (Ki) of 0.77 µm (Figure 7a,b). In vitro biotin synthesis assays, using a reporter strain MG1655 Δ*bioFCD* that accumulates red pigment proportional to biotin levels, confirmed the biotin synthesis mediated by BioE. Addition of 466982 reduced pigment production in a concentration‐dependent manner, yielding a Ki of 0.605 µm (Figure 7c,d). As biotin is essential for lipid metabolism, BioE inhibition by 466982 disrupts fatty acid synthesis and bacterial viability (**Figure** **7**e). On biotin‐limited media, colony counts of *E. coli* Δ*bioH* complemented with BioE and *E. meningoseptica* decreased with increasing 466982 concentrations (Figure 7f). These results establish 466982 as a promising antimicrobial lead targeting biotin biosynthesis, though structural optimization is warranted to enhance potency and bactericidal efficacy.

## 3 Discussion

BioE is a distinct class of ferritin‐like oxygenases that catalyzes the long‐chain acyl‐ACP/CoA substrates to generate pimeloyl moiety. This pathway diverges mechanistically from three established biotin synthesis routes: the canonical BioC‐BioH, BioI‐BioW, and BioZ pathways. Phylogenetic analyses revealed a broad distribution of the BioE pathway across bacterial lineages, including Weeksellaceae, Flavobacteriaceae, Chitinophagaceae, Sphingobacteriaceae, Flectobacillaceae, Burkholderiaceae, and intracellular parasitic Chlamydiaceae (Figure 1a). Notably, *bioE* frequently co‐occurred with *bioL* at genomic loci (Figure 2a), whereas Chlamydiaceae retained intact *bioE* loci but lacked *bioL* (Figure 1c). *C. trachomatis, C. caviae*, and *C. muridarum* have entirely lost their biotin biosynthesis gene clusters (Figure 1d; Figure S1, Supporting Information), an intriguing evolutionary trait. Functional complementation assays in *E. coli* deleted *bioH*, *bioC*, or *bioH*/*bioC* demonstrated that both EmBioE and CpBioE could bypass the BioC‐BioH pathway, underscoring their conserved functional roles (Figure 1e). BioL, a MocR‐family regulator, consists of an N‐terminal HTH DNA‐binding domain, a linker region, and a C‐terminal PLP‐dependent aminotransferase domain (Figure 2g).^[^ ^50^ ^]^ The conserved basic residue R50 in the HTH domain may mediate interactions with the promoter (Figure S3a, Supporting Information), and K340 in the C‐terminal domain serves as a canonical PLP‐covalent binding site (Figure S3, Supporting Information). Site‐directed mutations in BioL (R50A and K340A) phenocopied *bioL* deletion, upregulating the expression of biotin synthesis genes (Figure 2i; Figure S2e, Supporting Information). To rule out the possibility that EmBioL functions as an auxiliary protein similar to BsaP,^[^ ^5^ ^]^ we complemented the *E. coli* Δ*bioF* strain with EmBioF. When co‐complemented with BsBioW, EmBioF was found to recognize pimeloyl‐CoA in vivo, indirectly confirming that EmBioL is not an auxiliary protein of BioF (Figure S2c,d, Supporting Information). Recent studies have reported the presence of a BioWF fusion protein in *Corynebacterium amycolatum* (CaBioWF).^[^ ^51^ ^]^ In contrast to EmBioL, based on the known characteristics of the MocR family, we speculated that the aspartate aminotransferase domain of EmBioL lacks enzymatic activity like CaBioWF.^[^ ^50^ ^]^ Unfortunately, the inherent instability of EmBioL has hindered our further characterization of this protein. Paradoxically, *bioL* deletion in *Elizabethkingia* and *Chryseobacterium* resulted in biotin auxotrophy, a phenotype rescued by exogenous biotin supplementation (Figure 2d,e; Figure S2b, Supporting Information). This contradicts expectations, as loss of a negative regulator should enhance *bioE/F/A/D/B* expression and biotin production. The paradoxical biotin auxotrophic phenotype upon *bioL* deletion is unprecedented in other biotin regulatory systems. For example, the loss of the negative regulators BioQ and BioR in *Mycobacterium* and *Agrobacterium*, respectively, did not elicit biotin auxotrophy.^[^ ^43^, ^52^ ^]^ In *E. coli*, BirA is essential because of its dual function as a biotin synthesis regulator and a biotin ligase mediating biotin incorporation into proteins.^[^ ^53^ ^]^ In *Elizabethkingia*, BirA lacks a DNA‐binding domain and retains only the biotin ligase domain, indicating that EmBirA is not involved in regulatory functions. Thus, EmBioL serves as the primary negative regulator of biotin synthesis in *Elizabethkingia*. However, the reason that *bioL* deletion causes biotin auxotrophy in *Elizabethkingia* but not in *Chlamydia*, which naturally lacks *bioL*, remains unclear. We hypothesize that functional divergence between EmBioE and CpBioE may underlie this discrepancy.

Both EmBioE and CpBioE are diiron oxygenases that harbor a conserved diiron catalytic center analogous to cyanobacterial aldehyde deformylating oxygenase (cADO)^[^ ^54^, ^55^ ^]^ (Figure 3d,e). In CpBioE, residues E41, E68, H71, E140, E170, and H173 coordinated two Fe^3+^ ions to form the active site, and mutation of these residues abrogated enzymatic activity (Figure 3h). Structurally, EmBioE functions as a homodimer, whereas CpBioE is a monomer, similar to cADO, both belong to the diiron‐carboxylate superfamily of dioxygen‐activating enzymes.^[^ ^54^ ^]^ Unlike cADO, which catalyzed the release of formate from C_(n)_ fatty aldehydes to produce C_(n‐1)_ hydrocarbons,^[^ ^54^, ^56^ ^]^ BioE‐mediated inert C7‐C8 bond cleavage occurs in long‐chain acyl‐ACP, although its electron transfer mechanism remains unresolved. EmBioE and CpBioE exhibited different oligomeric states and substrate specificities. EmBioE functions as a homodimer capable of recognizing both long‐chain acyl‐ACP and acyl‐CoA, whereas CpBioE acts as a monomer, restricted to acyl‐ACP binding (Figure 4).

In *B. subtilis*, the heme‐dependent P450 enzyme BioI catalyzes the conversion of long‐chain acyl‐ACP to pimeloyl‐ACP, bypassing the *E. coli* BioC‐BioH pathway.^[^ ^29^, ^32^, ^57^ ^]^ Notably, *bioI* is genetically redundant in *B. subtilis*, as deletion of this gene does not impair biotin synthesis.^[^ ^29^, ^32^, ^47^, ^57^ ^]^ Althougth BioE and BioI share overlapping substrate and product profiles, they belong to distinct enzyme families: CpBioE is a non‐heme iron‐dependent oxygenase, whereas BioI is a heme‐dependent P450. Despite their divergent cofactor requirements, both enzymes are hypothesized to mediate fatty acid chain cleavage through alcohol and threo‐diol intermediates, highlighting convergent mechanistic strategies for generating biotin precursors across bacterial lineages.^[37,^ ^47^ ^]^Both CpBioE and EmBioE could rescue the growth of *E. coli* Δ*bioH* in biotin‐free M9 medium (Figures 1e and 5a). However, overexpression of EmBioE in wild‐type *E. coli* MG1655 imposed a fitness cost, manifested as growth retardation, whereas CpBioE did not (Figure 5b). Confocal microscopy revealed a significant increase in dead cells upon EmBioE overexpression, which was abolished by mutating the active‐site residue E26A; no such effect was observed with CpBioE (Figure 5d–g). Supplementation with stearic acid via the VhAasS fatty acid uptake system reversed growth arrest induced by EmBioE (Figure 5i), indicating that EmBioE overexpression depletes long‐chain fatty acids, disrupts the balance between fatty acid metabolism and biotin synthesis, and causes metabolic stress on the host cell. In *Elizabethkingia*, Δ*bioL* strains failed to grow in biotin‐restricted media, and supplementation with the biotin precursor DTB did not rescue this phenotype. In contrast, the Δ*bioL* Δ*bioE* double knockout strain regained viability upon DTB supplementation (Figure 5k), suggesting that unregulated EmBioE activity exacerbates metabolic perturbation. Transcriptomic analysis showed that *bioL* deletion upregulated the entire biotin biosynthesis operon, leading to excessive consumption of ATP/SAM (Figure 2i,j). The promiscuous substrate binding of EmBioE (recognizing both acyl‐ACP and acyl‐CoA), compared with the strict acyl‐ACP specificity of CpBioE, likely underlies the observed fitness cost. This may explain why *Elizabethkingia* requires the repressor BioL, whereas *Chlamydia* evolved without *bioL*. However, *Chlamydia* are primarily host‐parasitic, acquiring most of their energy and nutrients from the host cell. Through comparative analysis of host associations and genomes across eight different *Chlamydia* species, our study revealed that most *Chlamydia* genomes retain not only the *de novo* biotin biosynthesis pathway but also a conserved FAS II pathway and the biotin transport pathway (BioY) (Figure S1, Supporting Information). Collectively, these pathways may confer a survival advantage to host‐parasitic *Chlamydia* despite the absence of regulatory BioL.

*E. meningoseptica* is an emerging opportunistic pathogen of significant clinical concern, and is associated with severe infections, such as meningitis, bacteremia, and other life‐threatening syndromes.^[^ ^49^ ^]^ Notably, most clinical isolates exhibit multidrug resistance, demonstrating insensitivity to first‐line antimicrobial agents including carbapenems, polymyxins, and tetracyclines.^[^ ^49^, ^58^, ^59^ ^]^ This critical resistance profile underscores the urgent need to characterize novel antibacterial targets for therapeutic interventions. The biotin biosynthesis pathway has been validated as a validated druggable target for ESKPE pathogens (*Enterococcus*, *Staphylococcus*, *Klebsiella*, *Pseudomonas*, *Escherichia*) and pathogenic *Mycobacterium* species.^[^ ^10^, ^12^, ^13^, ^60^ ^]^ Here, we postulate that the BioE‐dependent biotin biosynthesis pathway represents a promising therapeutic target for such pathogens. Through in silico screening, the compound 466982 was identified as a specific binder in the substrate‐binding pocket of BioE. Structural analyses revealed interactions between 466982 and key residues within the lipid‐binding domain (L22, Y117, I124, and L131) (Figure 6a–c,f). SPR assays confirmed moderate binding affinity between BioE and 466982, whereas the BioE(L22A, Y117A, I124A, and L131A) mutants abrogated binding entirely (Figure 6d,e; Figure S12, Supporting Information). In vitro functional assays demonstrated that 466982 potently inhibited BioE‐mediated cleavage of the substrate C18‐ACP to generate pimeloyl‐ACP, as well as BioE‐dependent reconstituted biotin biosynthesis (Figure 7a–d). Antibacterial susceptibility testing showed that 466982 exerted growth‐inhibitory effects on *E. meningoseptica* strains (Figure 7f), supporting its potential as a lead antibacterial candidate. Collectively, these findings establish 466982 as a promising starting point for the development of BioE‐targeted antibiotics, with subsequent medicinal chemistry efforts focused on enhancing binding avidity and intracellular penetration. Notably, our study has several limitations that warrant consideration. The structures of BioE and its substrate‐bound complex presented herein were primarily predicted by AlphaFold3 and molecular docking simulations, which may exhibit discrepancies from the native 3D structures. Thus, additional efforts from multiple research teams will be required to experimentally resolve the high‐resolution crystal structure of BioE and elucidate its catalytic mechanism at the atomic level. Furthermore, the previously identified small‐molecule inhibitor targeting BioE, despite demonstrating robust enzymatic inhibitory activity, failed to exert significant antibacterial effects. This observation suggests that chemical modification or structural optimization of this compound is necessary to enhance its antimicrobial potency.

In summary, we identified BioE as a novel non‐heme Fe‐dependent oxygenase that catalyzes the cleavage of inert C7‐C8 bonds in long‐chain acyl groups to generate pimeloyl moiety, which is a critical precursor for biotin biosynthesis. The *bioE* gene exhibits a broad distribution across diverse bacterial species, including the intracellular parasite *Chlamydia*. However, *Elizabethkingia*‐derived EmBioE and *Chlamydia*‐derived CpBioE displayed distinct structural and biochemical features, highlighting genetic diversity within the BioE family. EmBioE showed dual functionality: it promoted bacterial growth under biotin‐restricted conditions by facilitating biotin synthesis, yet its overexpression imposed a fitness cost on host cells. In the Weksellaceae family, which includes *Elizabethkingia*, the repressor BioL is essential for precisely regulating EmBioE expression, and balancing biotin production with lipid metabolic demands. In contrast, *Chlamydia* has evolutionarily lost BioL may because of the strict substrate specificity of CpBioE, which may minimize cellular damage from unintended lipid metabolism perturbation. The BioE‐mediated biotin synthesis pathway represented a potential antimicrobial target. Our study demonstrates that the inhibitor 466982 potently suppresses BioE enzymatic activity and exhibits antimicrobial efficacy, positioning it as a promising lead compound for developing therapies against multidrug‐resistant *E. meningoseptica* infections.

## 4 Experimental Section

### RNA Extraction and Quantitative Real‐Time PCR (RT‐qPCR)

Overnight cultures were subcultured at a 1:100 ratio into 20 mL fresh TSB medium (with or without biotin) and grown at 37 °C with shaking at 180 rpm until reaching the exponential phase (OD600 = 0.6–0.8). Total RNA was extracted using AG RNAex Pro reagents (Accurate Biology, China) and purified with the RNeasy Mini Kit (Qiagen, USA). RNA purity and integrity were assessed by agarose gel electrophoresis and NanoDrop2000 spectrophotometry (Thermo Fisher Scientific, USA). cDNA synthesis was performed using the EvoM‐MLV RT Mix Kit (Accurate Biology, China) according to the manufacturer's instructions. The 16S rRNA gene served as an internal reference for normalizing expression levels across samples. Quantitative real‐time PCR (qPCR) was conducted using the Novostart SYBR qPCR SuperMix Kit (Novoprotein) on an Applied Biosystems ABI 7500 Sequence Detection System (Applied Biosystems, USA). Relative gene expression differences were calculated using the 2^−ΔΔCt^ method.^[^ ^61^ ^]^ Data were derived from at least three biological replicates.

### Protein Expression and Purification

The BL21(DE3) strain carrying pET28a‐EmBioE was used to isolate the 6x His‐tagged EmBioE enzyme, while the BL21(DE3) strain carrying pBAD24‐CpBioE was used for the 6x His‐tagged CpBioE enzyme. First, the strains were inoculated into 1 L of LB liquid medium and cultured to the logarithmic growth phase (OD_600_ ≈0.8). The BL21(DE3) pET28a‐EmBioE strain was induced with 0.1 mm isopropyl‐β‐D‐thiogalactopyranoside (IPTG) at 18 °C, and the BL21(DE3) pBAD‐CpBioE strain was induced with 0.2% L‐arabinose.^[^ ^26^ ^]^ Bacterial pellets were collected the next day, resuspended in lysis buffer (20 mm Tris‐HCl (pH 8.0), 300 mm NaCl, and 20 mm imidazole), and lysed via high‐pressure homogenization. The centrifuged supernatant was incubated with Ni‐NTA Beads at 4 °C for 1 h with rotation. Following affinity chromatography, impurities were removed using wash buffer (20 mm Tris‐HCl (pH 8.0), 300 mm NaCl, and 50 mm imidazole), and the target EmBioE was eluted with elution buffer (20 mm Tris‐HCl (pH 8.0), 300 mm NaCl, and 300 mm imidazole). The BioE protein samples were buffer‐exchanged into GF buffer (20 mm Tris‐HCl (pH 8.0), 300 mm NaCl) and analyzed by gel filtration using a Superdex 75 column (Cytiva).^[^ ^33^ ^]^ The purity of EmBioE and CpBioE in the target peaks was verified by 12% SDS‐PAGE.

*E. coli* BL21(DE3) cells harboring the plasmid pET28a‐Vh*aasS* were used for AasS expression.^[^ ^62^, ^63^ ^]^ A 1 L culture of this strain was induced overnight at 16 °C with 0.2 mm IPTG. Cells were harvested by centrifugation (6000 × g, 10 min, 4 °C), washed twice with lysis buffer (20 mm Tris‐HCl (pH 8.0), 300 mm NaCl), and then resuspended in the same lysis buffer for cell disruption. Following lysis by pressure homogenization, the lysate was clarified by centrifugation (15000 × g, 30 min, 4 °C) to remove insoluble debris. The clarified supernatant was applied to a Ni‐NTA agarose resin column (Smart‐lifesciences) to capture the N‐terminally 6×His‐tagged AasS protein. Contaminating proteins were removed by washing the column with lysis buffer supplemented with 20 mm imidazole. The target VhAasS protein was then eluted completely using lysis buffer containing 300 mm imidazole. Subsequently, the purity of the concentrated AasS protein was verified by 12% SDS‐PAGE, followed by Coomassie Brilliant Blue staining.

*E. coli* BL21(DE3) cells co‐harboring two plasmids (pET28a‐Ec*acpP* and pBAD24‐Ec*acpS*) were inoculated into LB medium supplemented with 50 µg mL^−1^ kanamycin (for pET28a‐Ec*acpP*) and 50 µg mL^−1^ ampicillin (for pBAD24‐*acpS*). The culture was grown at 37 °C with shaking until the optical density at 600 nm reached ≈0.8. For protein induction, the culture was cooled to 16 °C, and then 0.1 mm IPTG (to induce EcAcpP expression from pET28a) and 0.2% (w/v) L‐arabinose (to induce EcAcpS expression from pBAD24) were added. Induction was continued overnight at 16 °C. Cells were harvested from ≈3 L of induced culture by centrifugation (6000 × g, 10 min, 4 °C) and resuspended in lysis buffer (50 mm Tris‐HCl (pH 8.0), 150 mm NaCl, 10 mm MgSO_4_, 2 mm DTT). The cell suspension was disrupted using a pressure homogenizer, and the lysate was clarified by centrifugation (15000 × g, 30 min, 4 °C). To convert apo‐ACP to holo‐ACP, the clarified lysate was supplemented with 2 mm CoA trilithium salt and incubated at 37 °C for 3 h with gentle shaking. After incubation, the mixture was mixed with ice‐cold isopropanol at a 1:1 (v/v) ratio and centrifuged at 15000 × g for 30 min at 4 °C to precipitate impurities. The resulting supernatant was collected and dialyzed overnight at 4 °C against dialysis buffer (50 mm MES potassium salt (pH 6.1), 150 mm NaCl, 2 mm DTT) to remove isopropanol and excess CoA. The dialyzed sample was concentrated using centrifugal concentrators and then loaded onto a HiTrap Q HP anion exchange chromatography column (GE Healthcare). Protein purification was performed using 25 mm Tris‐HCl buffer (pH 8.0) with a linear NaCl gradient (150 mm to 1 m) to elute holo‐ACP. Fractions containing holo‐ACP were collected, pooled, and concentrated using Amicon Ultra centrifugal filters (Millipore) with a 3 kDa molecular weight cut‐off (MWCO).^[^ ^64^, ^65^ ^]^ Holo‐ACP was distinguished from its apo‐ACP via conformation‐sensitive urea gel electrophoresis.^[^ ^63^ ^]^

To purify soluble BioL protein, an expression system in *E. coli* BL21(DE3) cells harboring the pGEX‐GST‐SUMO‐BioL plasmid was established. The detailed experimental procedures are described as follows: Briefly, a 2 L culture of the recombinant *E. coli* strain was grown to the mid‐log phase and subsequently induced with IPTG at 30 °C for 4 h. After induction, bacterial cells were harvested by centrifugation and lysed using a French press. The resulting cell lysate was clarified via centrifugation, resuspended in 2×PBS, and then incubated with glutathione‐Sepharose resin for 1 h to enable the specific binding of the GST‐SUMO‐BioL fusion protein to the resin. Following removal of unbound proteins by extensive washing with 2×PBS, Ulp1 peptidase was added to the resin‐bound GST‐SUMO‐BioL fusion protein, and the mixture was incubated overnight at 4 °C to cleave the GST‐SUMO tag. The tag‐free BioL protein was then eluted using an elution buffer composed of 2×PBS (274 mm NaCl, 5.4 mm KCl, 16 mm Na_2_HPO_4_, 2.92 mm KH_2_PO_4_, (pH 7.4)), 1 mm pyridoxal 5′‐phosphate (PLP), and 5% (v/v) glycerol. The purity of the eluted BioL protein was assessed by 12%SDS‐PAGE. The purified BioL protein exhibited a faint yellow hue, a characteristic that may indicate its PLP‐bound form. Notably, the purified BioL protein showed inherent instability and a high tendency to precipitate during experimental manipulations.

### Electrophoretic Mobility Shift Assay (EMSA)

The electrophoretic mobility shift assay (EMSA) was employed to determine the binding ability of the BioL protein to promoter DNA probes. Two probes used in the experiment, designated as P*bioE* and P*bioB*, each correspond to an ≈50‐bp palindromic sequence and are located in the promoter regions of the *bioEFD* and *bioBA* genes, respectively (Table S2, Supporting Information). The EMSA reaction system had a total volume of 20 µL, with the buffer composition as follows: 20 mm Tris‐HCl (pH 8.0), 50 mm KCl, 5 mm MgCl_2_, 1 mm dithiothreitol (DTT), and 5% glycerol. The reaction was incubated at room temperature for 20 min. The mixture containing complexes formed by the DNA probes and the BioL regulator was electrophoretically separated using a 5% native polyacrylamide gel. Subsequently, the gel was stained with GelRed nucleic acid stain (Abclonal, China) for 15 min. Finally, the results were observed and documented using a UV transilluminator (SHENHUA, China).

### Detection of the Intracellular ATP and SAM Content

Intracellular ATP content of *Elizabethkingia* was determined using the ATP Assay Kit (WST‐1 Method) manufactured by mIbio (Cat. ml098821), following the protocol described below: *Elizabethkingia* wild‐type, Δ*bioL*, and CΔ*bioL* strains were first cultured in TSB medium until reaching the exponential phase (OD_600_ ≈ 0.7). Bacterial cells were harvested by centrifugation and washed three times with 1×PBS to remove residual medium. The washed cells of all three strains were then transferred to biotin‐depleted TSB medium, while an additional aliquot of the Δ*bioL* mutant was separately subcultured into regular TSB medium. All cultures were incubated for another 5 h to deplete residual intracellular biotin, after which bacterial cells were collected via centrifugation. For ATP extraction: Bacterial cells corresponding to 1×10^8^ colony‐forming units (CFU) were resuspended in 1 mL of extraction buffer. Cells were disrupted by ultrasonication under the following conditions: on ice, power output of 200 W, sonication cycles of 2 s (on) and 1 s (off), with a total processing time of 1 min. The lysate was centrifuged at 10000 × g and 4 °C for 10 min; the supernatant was transferred to a new microcentrifuge tube, mixed thoroughly with 500 µL of chloroform by vortexing, and centrifuged again at 10000 × g and 4 °C for 3 min. The resulting upper aqueous phase was collected and kept on ice until analysis. Finally, a visible spectrophotometer was set to a wavelength of 450 nm, and the absorbance of each sample was measured. The intracellular ATP content of *Elizabethkingia* cells in each group was calculated based on the measured absorbance values.

Intracellular S‐adenosyl methionine (SAM) content in *Elizabethkingia* cells was determined using the Microorganism S‐adenosyl Methionine (SAM) ELISA Kit (MEIMIAN, Cat. MM‐1111W1). Bacterial cells were processed following the same protocol as described for intracellular ATP determination. Collected cells were resuspended in 1×PBS, subjected to ultrasonication, and the resulting supernatant was collected for subsequent assay. The assay was performed as follows: Designate standard wells, test sample wells, and a blank well on the plate. Add 50 µL of the SAM standard solution to each standard well. Add 10 µL of the collected supernatant (test sample) to each test sample well, followed by 40 µL of Sample Diluent; no reagents were added to the blank well. Add 100 µL of HRP‐conjugated reagent to each well (excluding the blank well, if specified by the kit; otherwise, follow kit instructions). Cover the plate with an adhesive strip and incubate at 37 °C for 60 min. Aspirate the liquid from each well, then perform a washing step; repeat this aspiration‐washing cycle four times, resulting in a total of five washes. For washing, fill each well with 400 µL of Wash Solution using a squirt bottle. Complete removal of residual liquid at each step is critical for ensuring assay accuracy. After the final wash, remove any remaining Wash Solution by aspiration or decantation. Invert the plate and blot it against clean paper towels to eliminate excess liquid. Add 50 µL of Chromogen Solution A and 50 µL of Chromogen Solution B to each well. Gently mix the plate by tapping its edge, then incubate at 37 °C for 15 min while protecting the plate from light. Add 50 µL of Stop Solution to each well; a color change from blue to yellow should be observed in positive wells. If the color appears green or the color change is non‐uniform, gently tap the plate to ensure thorough mixing. Measure the optical density (OD) at 450 nm using a microtiter plate reader within 15 min of adding the Stop Solution. Finally, the SAM content in different *Elizabethkingia* strains (WT, Δ*bioL*, CΔ*bioL*, and Δ*bioL+*biotin strains) was calculated based on the standard curve generated from the SAM standard solutions.

### Spectroscopic Characterization of BioE

UV–visible spectroscopic characterization of the BioE and BSA (Bovine serum albumin) protein (NO. C500642, Sangon Biotech (Shanghai)) was conducted using EVOLUTION 600 (Thermo Fisher Scientific, USA). Buffer was used as a blank control, and the spectra of the EmBioE, CpBioE, and BSA were recorded in the 240–600 nm wavelength range.^[^ ^56^ ^]^ The protein concentrations were determined using a NanoDrop 2000 spectrophotometer (Thermo Fisher Scientific, USA) based on the absorbance at 280 nm, with extinction coefficients calculated by the ExPasy‐ProtParam (https://web.expasy.org/protparam/).

### Determination of Iron Content By Inductively Coupled Plasma‐Mass Spectrometry (ICP‐MS)

Iron content in CpBioE was quantified by inductively coupled plasma‐mass spectrometry (ICP‐MS). For sample preparation, 20 µm protein solutions were mixed with an equal volume of trace‐metal‐grade concentrated nitric acid (Thermo Fisher Scientific, USA) and subjected to heat digestion at 100 °C for 30 h. Following digestion, samples were diluted to a final concentration of 2% (v/v) nitric acid with Milli‐Q water and filtered through a 0.22 µm syringe filter. Elemental analysis was performed using an Agilent Technologies 7800 ICP‐MS instrument.^[^ ^56^, ^66^ ^]^ Targeted isotopes for quantification included Fe, Mn, and Zn. All data represent mean values from three independent biological replicates (*n* = 3).

### Substrate Recognition by BioE

To assess BioE's recognition of long‐chain acyl‐CoA substrates, product formation was monitored at 260 nm.^[^ ^30^, ^31^ ^]^ The 100 µL reaction mixture comprised 20 mm Na‐HEPES (pH 7.5), 100 mm NaCl, 2 mm MgCl_2_, 0.2 mm DTT, 0.3 mm C18‐CoA, 75 mm PMS, 750 mm NADH, and 30 mm BioE protein. Reactions were incubated at 30 °C for 1 h, quenched with methanol (final concentration 50%), and quick‐frozen in liquid nitrogen. Following thawing, precipitated proteins were removed by centrifugation (10 min), and 100 µL of supernatant was analyzed by HPLC using a Shimadzu Prominence LC‐20A system with a C18 analytical column. The mobile phase consisted of Solvent A (50 mm ammonium acetate, pH 5) and Solvent B (100% methanol), with an elution profile of 0% B for 5 min, linear gradient to 70% B over 35 min, further gradient to 100% B over 5 min, and isocratic elution at 100% B for 10 min (flow rate: 1 mL min^−1^, room temperature). The product pimeloyl‐CoA was identified by a distinct peak at 260 nm.

### Enzymic Analysis of BioE by Urea‐PAGE

Following the reaction conditions established for cADO and BioH,^[^ ^21^, ^55^ ^]^ the enzymatic activity of BioE in vitro was reconstituted. The 50 µL reaction mixture contained 20 mm Na‐HEPES (pH 7.5), 100 mm NaCl, 0.2 mm DTT, 75 µm PMS, 750 µm NADH, 5% glycerol, 150 µm long‐chain acyl‐ACP substrate, and 30 µm BioE protein. After incubation at 30 °C for 1 h, 15 µL of the reaction mixture was resolved by 0.5 m urea/17.5% PAGE (pH 9.5, 130 V for 2.5 h) under conformation‐sensitive conditions. In this urea‐gel system, the product C7‐ACP was distinguishable from the C18‐ACP substrate by its slower migration rate. To determine the inhibition constant of 466982 against BioE, different concentrations of inhibitor 466982 were added to the reaction system, and the reduction in product pimeloyl‐ACP was detected via relative quantification to assess the inhibitory effect.

### Surface Plasmon Resonance (SPR)

SPR experiments were performed using a Biacore 1K system (Cytiva) equipped with a CM5 sensor chip (carboxymethylated dextran matrix).^[^ ^67^ ^]^ The BioE protein was covalently immobilized onto the sensor surface via amine coupling chemistry following the manufacturer's protocol. Briefly, the chip surface was activated with a 1:1 mixture of N‐ethyl‐N′‐(3‐dimethylaminopropyl) carbodiimide hydrochloride (EDC) and N‐hydroxysuccinimide (NHS) for 7 min at a flow rate of 10 µL min^−1^. The BioE protein, diluted to 20 µg mL^−1^ in 10 mm sodium acetate buffer (pH 5.0), was injected until a target immobilization level of ≈5000 response units (RU) was achieved. Residual active groups were blocked with 1 m ethanolamine‐HCl (pH 8.5). The ligand was serially diluted in running buffer (HBS‐EP+: 10 mm HEPES, 150 mm NaCl, 3 mm EDTA, 0.05% (v/v) Surfactant P20, pH 7.4) to generate eight concentrations: 100, 50, 25, 12.5, 6.25, 3.125, 1.56, and 0.78 µm. Each concentration was injected over the protein‐immobilized surface and a reference surface (activated/blocked but without protein) in single‐cycle kinetics mode at 25 °C. Association and dissociation phases were monitored for 120 s each at a flow rate of 30 µL min^−1^. Surface regeneration was achieved by injecting 10 mm glycine‐HCl (pH 2.0) for 30 s.

Sensorgrams were double‐referenced by subtracting both the reference surface response and the buffer injection response. Kinetic parameters (association rate constant k_on_, dissociation rate constant k_off_, and equilibrium dissociation constant KD) were determined by globally fitting the data to a 1:1 Langmuir binding model using Biacore Insight Evaluation Software (Version 3.2). All experiments were performed in triplicate.

### Confocal Microscopy and Biofilm Formation

Overnight cultures of *E. meningoseptica* wild‐type (WT), Δ*bioL* mutant, and complemented strain (CΔ*bioL*) were inoculated at a 1:100 dilution into tryptic soy broth (TSB) supplemented with or without biotin. Cultures were grown to mid‐logarithmic phase (OD_600_ = 0.3) before 1 mL of each was transferred to a Cellvis 4‐chamber glass‐bottom dish (P35G‐1.5‐14‐C). Biofilms were formed via static incubation at 37 °C for 24 h. After incubation, the supernatant was gently aspirated using a 1 mL insulin syringe, and adherent cells were washed three times with 1 mL pre‐warmed 1× phosphate‐buffered saline (PBS) to remove non‐adherent bacteria. Cells were stained with SYTO9 (Thermo Fisher Scientific, USA) diluted 1:1000 in 1× PBS (final concentration 5 µm) for 20 min at room temperature in the dark, followed by two gentle washes with 1× PBS to remove excess dye.^[^ ^68^ ^]^

Different strains of *Elizabethkingia* were prepared as follows: the overnight‐cultured bacterial suspensions were diluted at a ratio of 1:1000 in fresh low‐biotin Tryptic Soy Broth (TSB) medium. Then, 200 µL of the diluted bacterial suspension was pipetted into each well of a 96‐well polystyrene plate. After static incubation at 37 °C for 48 h, each well was rinsed with water, and the biofilms were subsequently fixed with 99% methanol. Thereafter, 200 µL of 0.1% crystal violet was added to stain the biofilms for 10 min. After removing the supernatant, the plates were rinsed three times with water. Once the crystal violet was dissolved with 200 µL of 95% ethanol, the optical density (OD) at a wavelength of 570 nm was measured.^[^ ^69^ ^]^ Statistical differences were analyzed using GraphPad Prism7 via unpaired two‐tailed Student's t‐test.

*E. coli* strains MG1655, MG1655::CEmBioE, MG1655::CEmBioE(E26A), and MG1655::CCpBioE were individually inoculated into 2 mL of M9 minimal medium supplemented with appropriate antibiotics (M9 salts, 2 mm MgSO_4_, 0.1 mm CaCl_2_, 0.4% glycerol, 0.2% casamino acids). Cultures were grown at 37 °C with shaking (220 rpm) to mid‐logarithmic phase (OD_600_ = 0.4). Gene expression was induced by adding L‐arabinose to a final concentration of 0.2%, followed by further incubation for 4 h. Post‐induction, 1 mL of each culture was transferred to a 1.5 mL microcentrifuge tube, centrifuged at 5000 ×g for 5 min at 4 °C, and the supernatant discarded. The bacterial pellet was washed twice by resuspension in 1 mL of 1× phosphate‐buffered saline (PBS, pH 7.4) followed by centrifugation. The final pellet was resuspended in 100 µL of 1× PBS. Dead/Live staining solution (Thermo Fisher Scientific, USA) was added at diluted 1:1000 in 1× PBS, final concentrations 5 µm SYTO9 and 5 µg mL^−1^ PI, and the mixture was incubated in the dark for 20 min at room temperature.^[^ ^70^, ^71^ ^]^ Following staining, 10 µL of the bacterial suspension was placed on a microscope slide pre‐coated with 1% agarose (prepared in 1× PBS). A coverslip was gently placed over the sample to prevent drying. Samples were immediately imaged using confocal microscopy.^[^ ^70^, ^72^ ^]^ Confocal imaging was performed using a Zeiss LSM 900 confocal microscope equipped with an Airyscan 2 detector and a Plan‐Apochromat 63×/1.40 oil immersion lens (DIC M27 objective). SYTO9 was excited at 488 nm using an argon laser, PI was excited at 561 nm using an argon laser. Z‐stack images were acquired in Airyscan mode with “Best Signal” processing, a pixel size of 66 nm (super‐resolution mode, 1834×1834 pixels), and a *z*‐axis step distance of 0.1 µm. Each sample was analyzed across three independent biological replicates. Post‐acquisition image processing and analysis were conducted using ZEN 3.4 (Blue Edition).

### Gas Chromatography Mass Spectrometry (GC‐MS)

*E. coli* MG1655 and MG1655 pEmBioE were inoculated into biotin‐deficient M9 minimal salt medium and cultured overnight. The following day, cultures were subcultured at a 1:100 dilution ratio into 50 mL of fresh biotin‐deficient M9 minimal salt medium. Upon reaching early exponential phase (OD_600_ ≈ 0.3), L‐arabinose was added to a final concentration of 0.2% to induce gene expression. Once *E. coli* MG1655 cultures reached OD_600_ ≈ 0.7, bacterial cells were harvested via centrifugation at 4200 × g and 4 °C for 15 min. Collected cells were washed three times with 1×PBS to remove residual medium, and bacterial pellets were collected and subjected to lyophilization, and then to weight. Lyophilized cells were resuspended in 1 ml of Solution I (3.75 m NaOH and 50% methanol [v/v]). After gentle mixing, the suspension was incubated in a 100 °C water bath for 35 min to complete fatty acid saponification. Samples were cooled to 80 °C, and 2 mL of Solution II (6 N HCl:methanol = 325:275 [v/v]) was added; following mixing, samples were incubated for 10 min to achieve fatty acid methylation. Subsequently, 1.25 mL of Solution III (n‐hexane:methyl tert‐butyl ether = 1:1 [v/v]) was added, and the mixture was vortexed to homogenize before being allowed to stand for phase separation. The organic phase was collected, washed with Solution IV (containing 300 mm NaOH), and transferred to a liquid‐phase vial. The organic phase was dried under a stream of nitrogen to yield fatty acid methyl ester samples, which were subsequently analyzed via gas chromatography‐mass spectrometry (GC‐MS).^[^ ^73^ ^]^

### Reconstitution of BioE‐Initiated Biotin Synthesis In Vitro

Following the method of Lin and colleagues with minor adjustments,^[^ ^21^ ^]^ a cell‐free crude extract of *E. coli* MG1655 Δ*bioH* was prepared, which provides the enzyme system involved in the late pathway of biotin synthesis. Briefly, the *E. coli* MG1655 Δ*bioH* strain was cultured in 200 mL of M9 defined medium supplemented with 1 nm biotin at 37 °C to mid‐log phase (OD_600_ = 0.8). After two washes with 20 mL of M9 medium to remove residual biotin, the cells were transferred to 1 L of biotin‐free M9 minimal medium and cultured at 37 °C for more than 5 h to induce intracellular biotin depletion. Subsequently, the collected STL96 cells were lysed twice by a French pressure cell, centrifuged at 16 800 rpm for 0.5 h at 4 °C. The supernatant was precipitated with 85% saturated ammonium sulfate, followed by centrifugation at 12 800 rpm for 1 h. Assuming that ACP molecules and small molecules are soluble in this solution, the dissolved precipitate was dialyzed overnight against 2 L of PBS buffer at 4 °C to remove ammonium sulfate and residual small molecules. The concentration of the STL96 crude extract was determined by NanoDrop (Thermo Fisher Scientific), and the final storage solution had a concentration of ≈175 mg mL^−1^.

Next, the BioE‐guided DTB/biotin synthesis system (total volume 200 µL) contained the following components: 5 mg of MG1655 Δ*bioH* crude extract, 30 µm of BioE enzyme, 100 µm C18‐ACP substrate, 0.1 µm alanine, 0.01 µm pyridoxal 5′‐phosphate (PLP), 0.1 µm glucose‐6‐phosphate, 0.1 µm NADPH, 0.1 µm NADH, 0.2 µm SAM, 0.1 µm ATP, 1.0 mm MgCl_2_, 0.1 µm KHCO_3_, 0.5 µm DTT. Typically, the reaction was maintained at 37 °C for ≈3 h, and terminated by treatment in a 100 °C metal bath for 10 min. The reaction mixture was centrifuged at 12 000 rpm for 10 min, and the supernatant containing DTB/biotin products was collected, and stored at −20 °C until use in biological assays. To prepare biotin‐indicating M9 agar plates, the biotin‐dependent MG1655 Δ*bioFCD* strain was used as a reporter strain to sense the presence of extracellular biotin/DTB. Briefly, ER90 was cultured overnight in 5 mL of M9 chemically defined medium containing 2 nm biotin, harvested by centrifugation at 6000 rpm for 6 min, washed twice with M9 medium, and transferred to 100 mL of biotin‐free M9 minimal medium. A 5 h starvation treatment was assumed to deplete intracellular biotin/DTB metabolites in ER90 cells. The cells were collected, washed twice, resuspended in 1 mL of biotin‐free PBS buffer, and stored at 4 °C for later use. Typically, the prepared MG1655 Δ*bioFCD* cells (100–150 µL) were mixed with 100 mL of melted M9 agar medium (≈55 °C) containing 0.01% (w/v) 2,3,5‐triphenyltetrazolium chloride (TTC) as a bacterial respiration indicator. Finally, four‐zone petri dishes were prepared, with each zone containing 4–5 mL of M9 agar medium overlaid with filter paper discs. Biotin/DTB spotted on the filter paper discs could sustain the survival of the MG1655 Δ*bioFCD* strain, thereby reducing TTC to form insoluble red formazan deposited around the colonies, while the indicator strain showed no growth signal in the absence of biotin.^[^ ^13^ ^]^ In the reaction system, different serial concentrations of small‐molecule inhibitors were added to suppress BioE activity, leading to reduce in vitro biotin synthesis and a smaller diameter of the red circle indicator. The inhibition constant of 466982 against BioE was determined based on the diameter of the red circle indicator.

### Virtual Screening the Inhibitors for BioE

Initially, the target protein was rigorously prepared to ensure a biologically reasonable conformation and compliance with PDB format specifications for subsequent docking and molecular dynamics analyses. The TopScience Refine Set virtual screening library (comprising 7172703 compounds) was employed, which had undergone prefiltering based on key physicochemical properties: molecular weight (MW, 300–800 Da), topological polar surface area (TPSA, 0–140 Å^2^), octanol‐water partition coefficient (LogP, 1–3), and water solubility (LogS, −4–1.5). High‐throughput molecular docking was first performed using KarmaDock, from which the top 10000 compounds with the highest initial docking scores were shortlisted. Subsequently, high‐precision docking was conducted via CarsiDock. All docking outcomes were re‐evaluated using the RTMScore scoring function and subjected to conformational optimization. Clustering analysis was then applied to identify the top 1000 compounds with the most favorable binding poses. Finally, molecular dynamics simulations were performed on these selected conformations to assess structural stability. Binding free energies were calculated using the molecular mechanics/generalized Born surface area (MM/GBSA) method, and the results were manually curated to eliminate false positives, yielding a final set of 200 most promising small‐molecule candidates. Among these, the top 10 compounds were purchased from TargetMol and used to test their binding affinity to BioE as well as their inhibition activity against BioE.

### Statistical Analyses

All statistical analyses were performed using GraphPad Prism 8.0. Statistical significance was assessed by an unpaired two‐tailed Student's *t*‐test. Statistically significant results in all figures are indicated as *: *p* < 0.05; **: *p* < 0.01; ***: *p *< 0.001; and ****: *p* < 0.0001.

## Conflict of Interest

The authors declare no conflict of interest.

## Author Contributions

M.Z, Y.F, X.Y, and Q.Q contributed equally to this work. Y.X, Z.R, and M.Z designed research and supervised the project; Y.X, M.Z, Y.F, X.Y, Q.Q, X.S, J.F, Y.K and Q.H performed research; M.Z, Y.F, X.Y, and Q.Q analyzed data; Y.X, Z.R, and M.Z wrote the paper.

## Supporting information



Supporting Information

## Data Availability

The data that support the findings of this study are available in the supplementary material of this article.
