# Supplementary material for: Balanced Expression of the Diiron Oxygenase BioE Is Essential for Biotin Homeostasis in Elizabethkingia meningoseptica
Source: Adv Sci (Weinh). 2025 Dec 12;13(10):e10491. doi: 10.1002/advs.202510491 (PMC12915131; doi:10.1002/advs.202510491)
Supplement: Supplementary file 1 — Supporting Information [file ADVS-13-e10491-s001.docx]

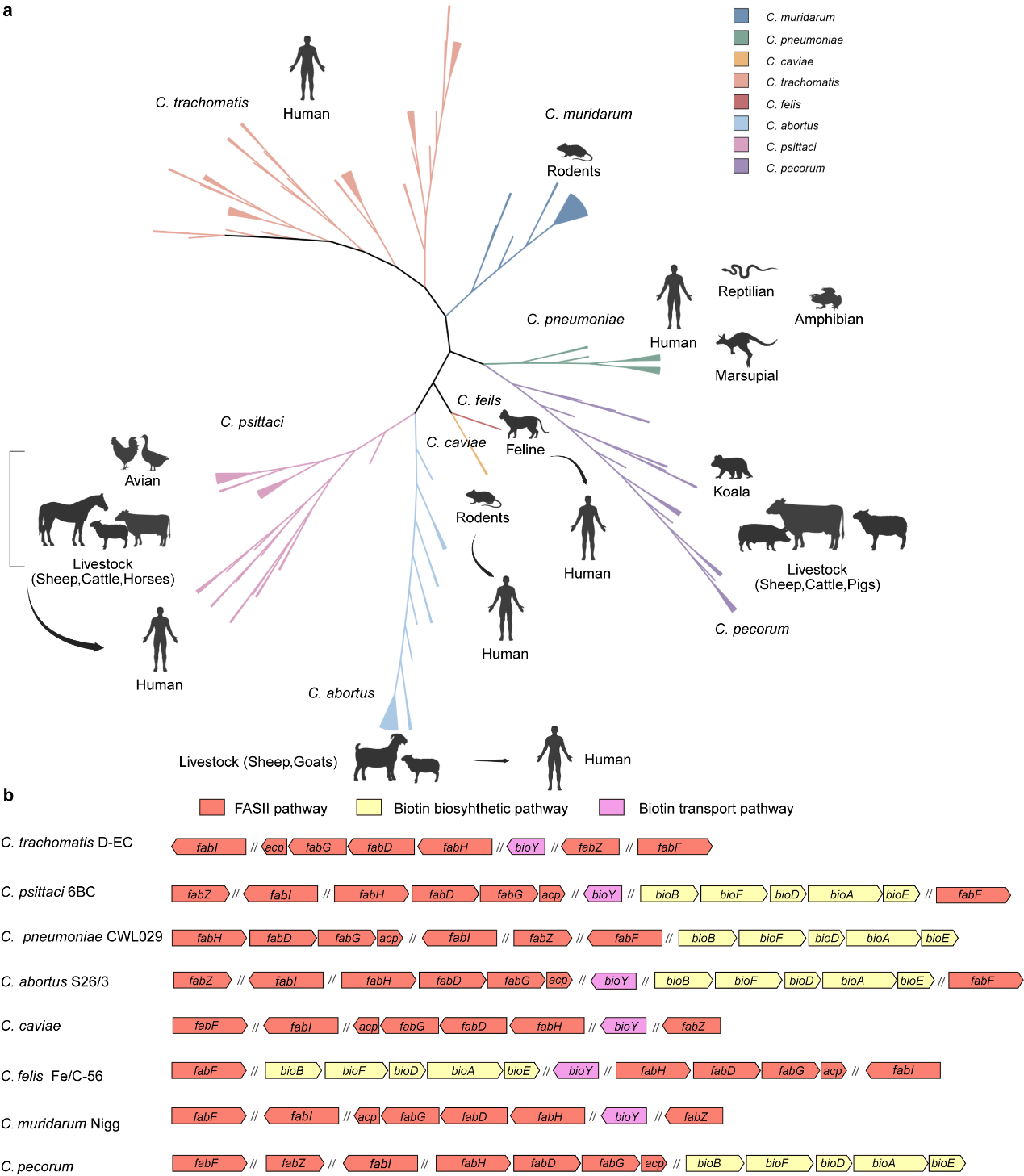


**Figure S1 Assessing associations between hosts of *Chlamydia* species and genetic context of the biotin metabolic pathway**

**a**. Genetic evolutionary relationships and hosts of eight *Chlamydia* species

**b**. Biotin-related metabolic pathways in *Chlamydia* species

FabD, FabH, FabF, FabG, FabZ, FabI, and AcpP are primarily key enzymes in the type II fatty acid metabolic pathway (FASII), which can provide substrate molecules (LC acyl-ACP) for biotin synthesis. BioE, BioF, BioA, BioD, and BioB are proteins involved in the biotin biosynthesis pathway; BioY is a biotin transporter protein that can uptake exogenous biotin into *Chlamydia* cells.

**Designations**: FAS II, type II fatty acid synthesis pathway


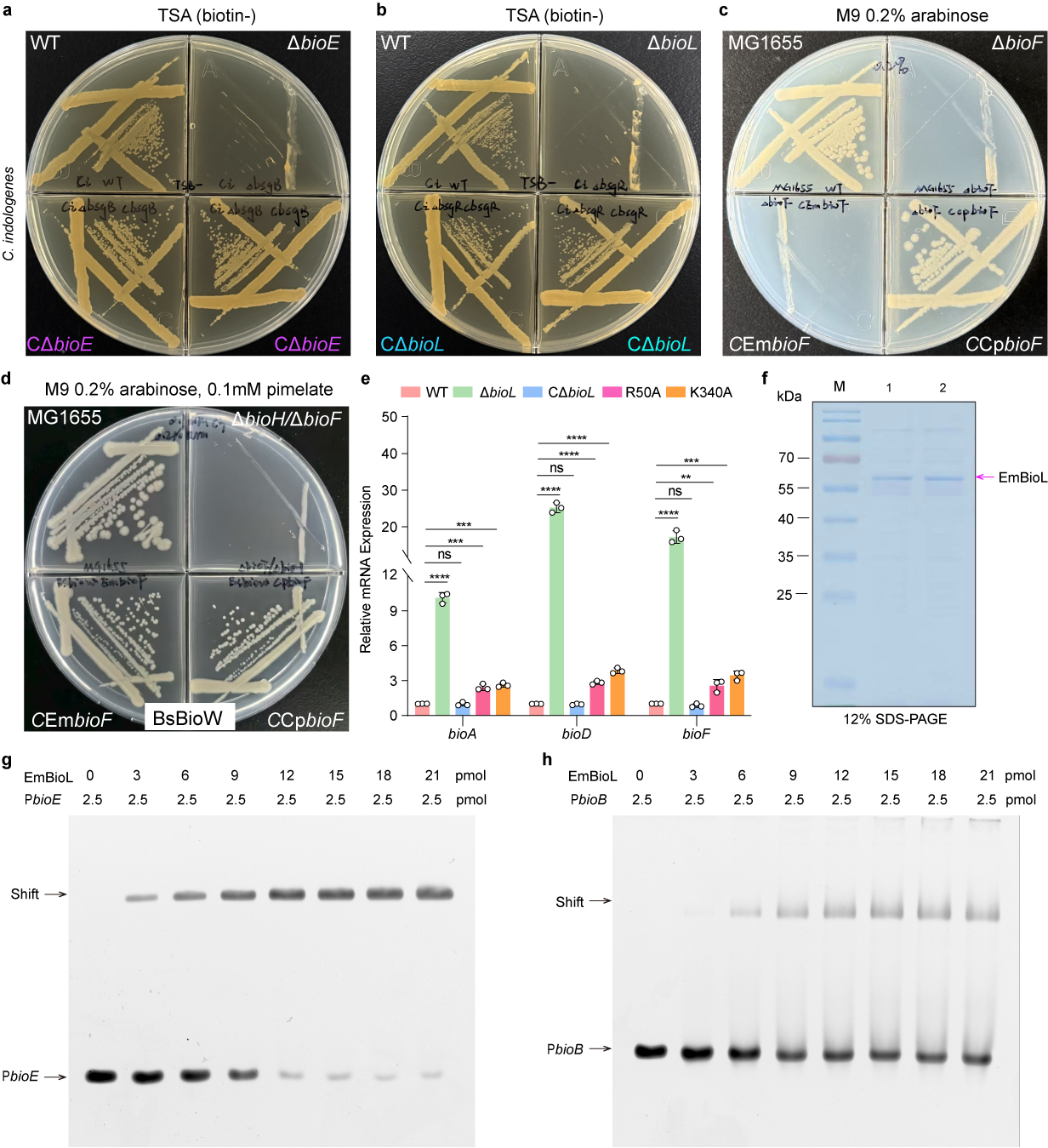


**Figure S2 Deletion of EmBioL results in a biotin auxotrophic phenotype**

**a.** Deletion of *bioE* resulted in growth arrest on biotin-free TSA medium, which was fully rescued by genetic complementation with the *bioE*

**b**. Disruption of *bioL* displayed a biotin auxotrophic phenotype in *E. meningoseptica*

**c**. CpBioF could complement the biotin auxotrophic phenotype of *E. coli* Δ*bioF*, whereas EmBioF could not

**d**. Simultaneous complementation of *E. coli* Δ*bioF* with both EmBioF and BsBioW restored its biotin auxotrophic phenotype

**e**. RT-qPCR analysis showed that mutations in BioL(R50A) and BioL(K340A) led to increased expression levels of the biotin biosynthesis gene cluster

Data represent mean ± SD from n = 3 biological replicates. The statistical significance was determined by unpaired two-tailed Student’s t-test (**P < 0.01, ***P < 0.001, ****P < 0.0001).

**f**. SDS-PAGE analysis demonstrated the purity of the BioL

**g-h**. EMSA experiments confirmed that BioL binds to the *bioE* and *bioB* promoters of the biotin biosynthesis gene cluster. The experiments were performed in three replicates, and one representative result is shown.


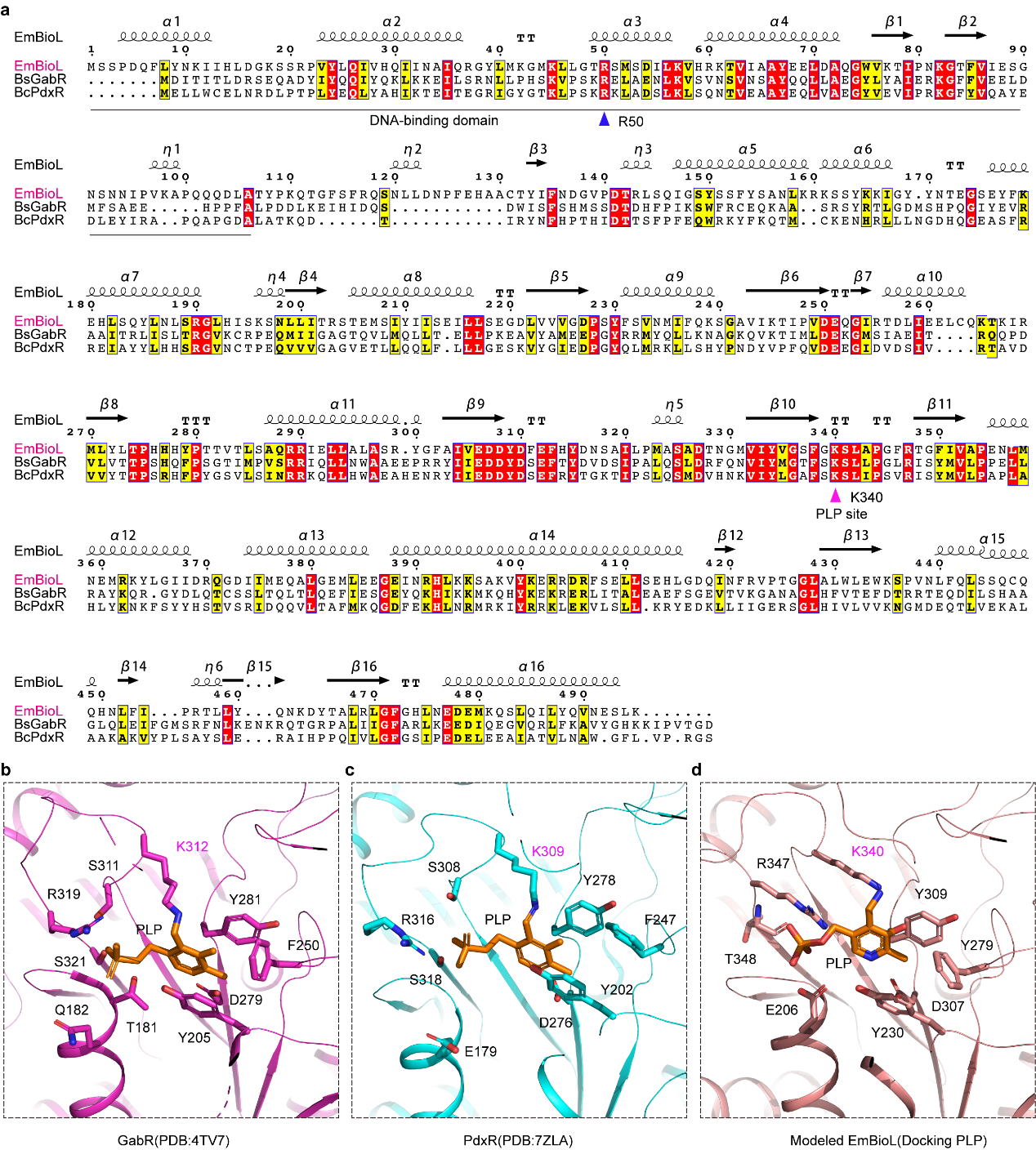


**Figure S3 EmBioL is a transcriptional regulator belonging to the MocR family**

**a**. Sequence alignment reveals that EmBioL shares conservation with the well-characterized BsGabR and BcPdxR

BsGabR and BcPdxR are well-studied transcriptional regulators in the MocR family: BsGabR controls γ-aminobutyric acid (GABA) metabolism in *Bacillus subtilis*, while BcPdxR regulates pyridoxal 5'-phosphate (PLP) metabolism in *Bacillus* *clausii*. R50 is a conserved amino acid residue in the helix-turn-helix (HTH) motif, indicated by a blue triangle; K340 is the covalent PLP-binding site in the AAT domain, denoted by a red triangle.

**b-d**. Simulated structural comparisons illustrate the conserved residues of EmBioL that interact with PLP

The structures of GabR (PDB: 4TV7) and PdxR (PDB: 7ZLA) have been resolved, whereas the structure of EmBioL was predicted using AlphaFold3. Structural comparisons revealed that GabR covalently binds a PLP molecule at position K312, and PdxR covalently binds a PLP molecule at position K309. Docking results suggested that EmBioL is likely to bind a PLP molecule at position K340. Furthermore, multiple conserved residues in these three proteins interact with PLP via hydrogen bonding and hydrophobic interactions.


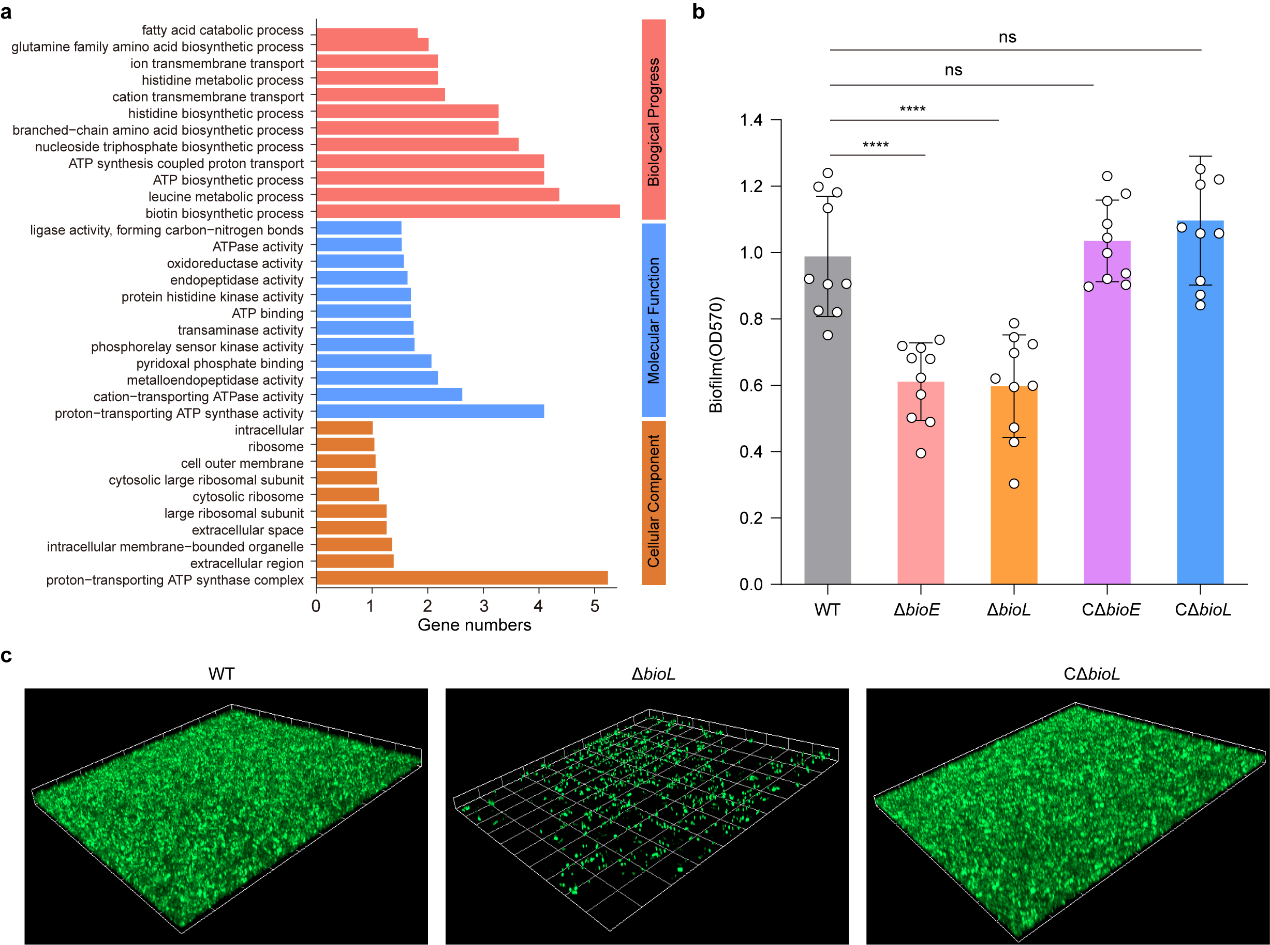


**Figure S4 Biofilm formation was impaired by the deletion of *bioL* in *E. meningsepticum***

**a**. Active energy metabolism under biotin-depleted conditions in *Elizabethkingia*

Transcriptome analysis revealed significant upregulation of ATP metabolism-related genes in *Elizabethkingia* grown without biotin TSA medium.

**b**. *bioL* knockout reduces biofilm production

Deletion of *bioL* significantly decreased biofilm biomass, representing a marked reduction compared to the wild-type strain. Complementation with both *bioE* and *bioL* restored biofilm biomass to wild-type levels.

The values are given as the means ± SD (n=10). Statistical significance was determined by unpaired two-tailed Student’s t-test (**P < 0.01, ***P < 0.001, ****P < 0.0001).

**c**. Confocal fluorescence microscopy confirms reduced biofilm formation in the *bioL* mutant

Visualization by confocal microscopy demonstrated decreased biofilm architecture in the *bioL* mutant strain relative to the wild-type.


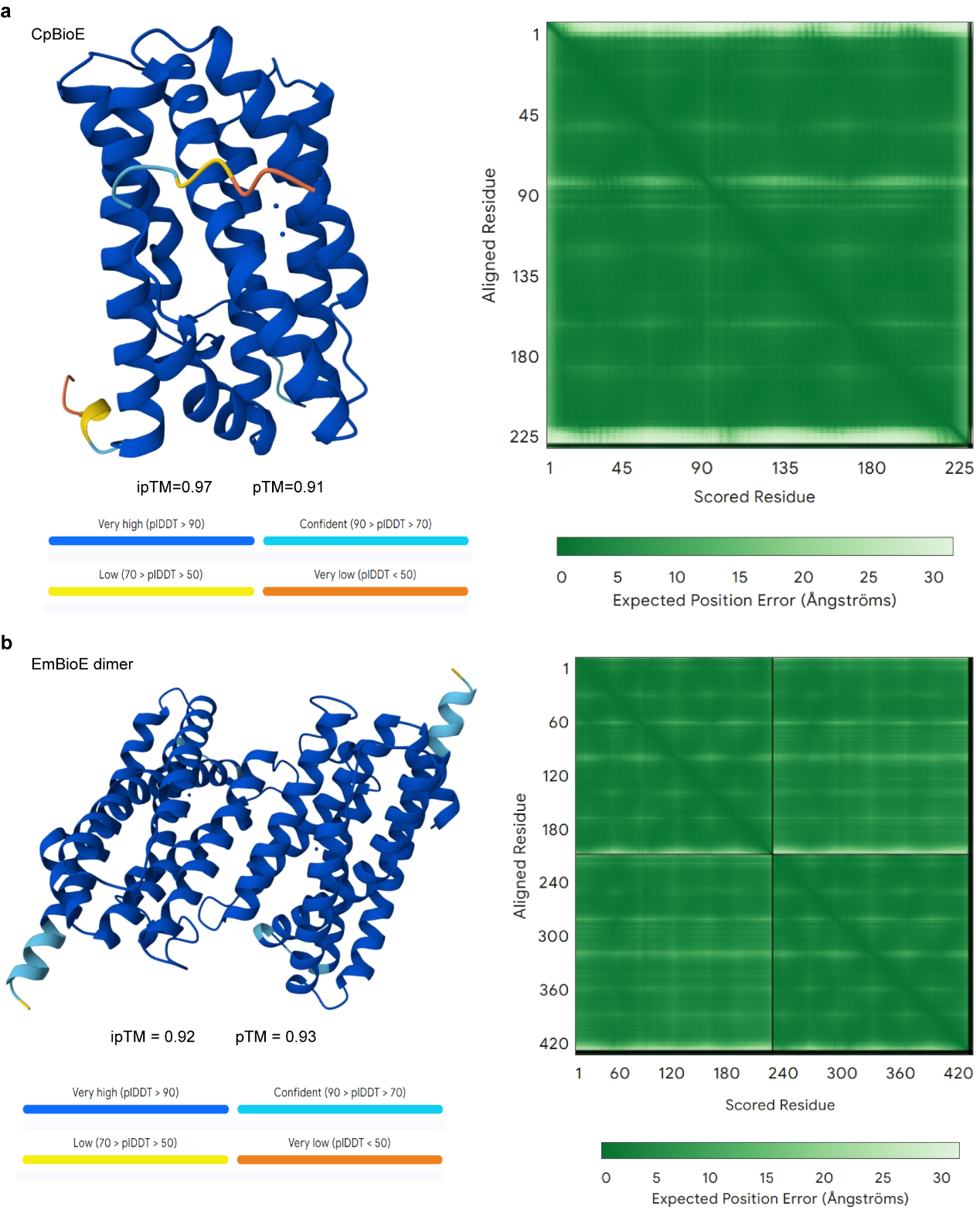


**Figure S5 High-confidence structures of CpBioE and EmBioE were generated by AlphaFold3**

**a**. The structure of CpBioE interacting with two iron atoms was generated by AlphaFold3

**b**. The simulated structure of the EmBioE dimer was produced via AlphaFold3

Both models exhibit ipTM and pTM values greater than 0.9, which are generally recognized as indicators of high structural confidence


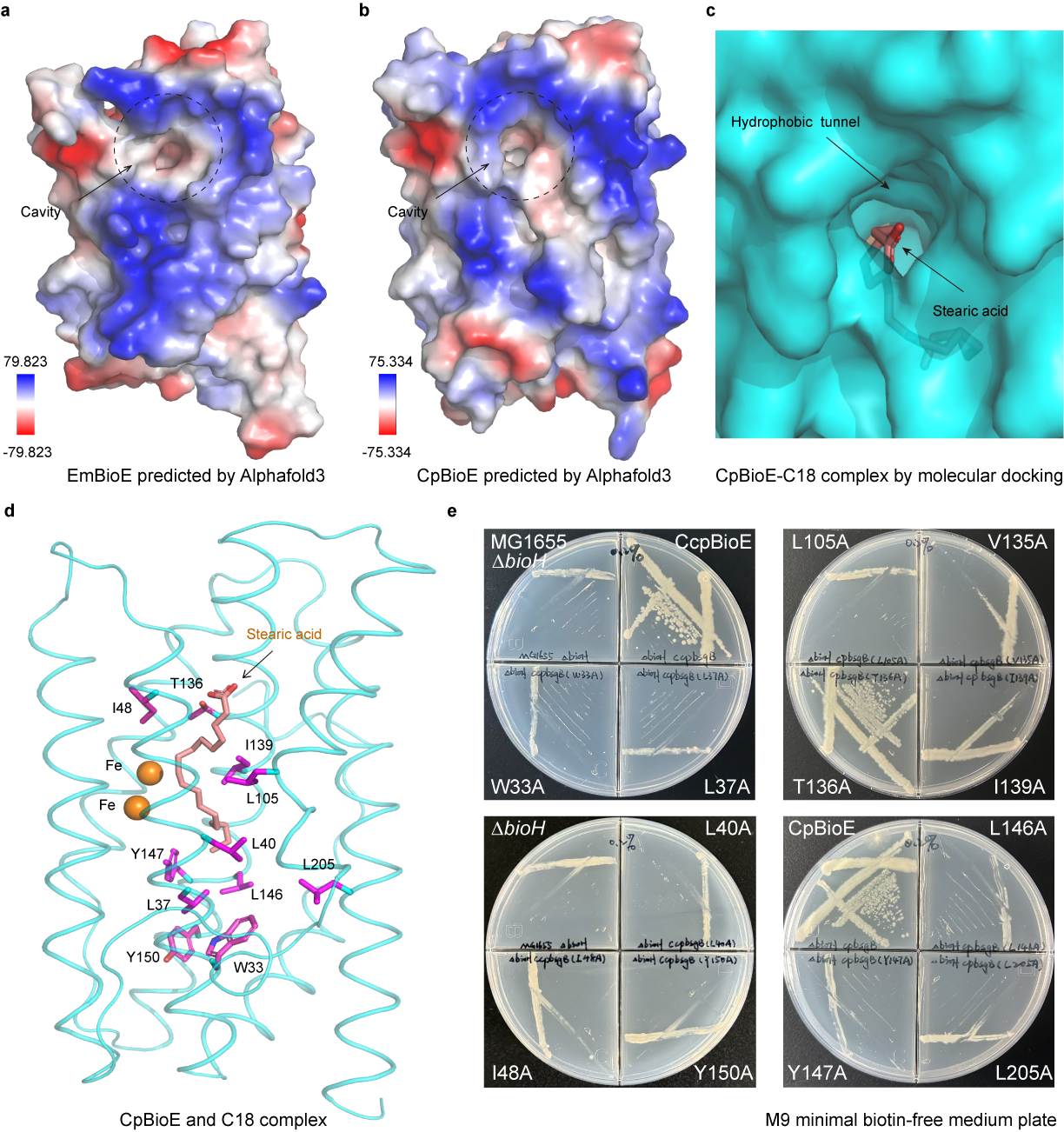


**Figure S6 Structural features of long-chain acyl group-binding pocket in CpBioE**

**a**. The vacuum electrostatic map of the EmBioE

**b**. Surface charge mapping of CpBioE reveals a hydrophobic pocket

A hydrophobic pocket in CpBioE is highlighted by circles and arrows.

**c**. Molecular docking demonstrates stearic acid binding to the hydrophobic pocket of CpBioE

**d**. Predicted key residues mediating interactions between CpBioE and stearic acid

Stearic acid is shown in a stick model, docked into the hydrophobic pocket of CpBioE, with its binding position highlighted by an arrow. AlphaFold3-based docking results identify a hydrophobic channel composed of W33, L37, L40, I48, L105, V135, T136, I139, Y147, and L205, which interacts with stearic acid via hydrophobic forces.

**e**. Site-directed mutagenesis validates the role of stearic acid-interacting residues in CpBioE function

Functional validation using selective media revealed that mutation of nine amino acids (W33, L37, L40, I48, L105, V135, I139, Y147, L205) abrogated BioE activity. A representative figure was selected from three replicates for presentation. All structures of both EmBioE and CpBioE are generated from AlphaFold3.


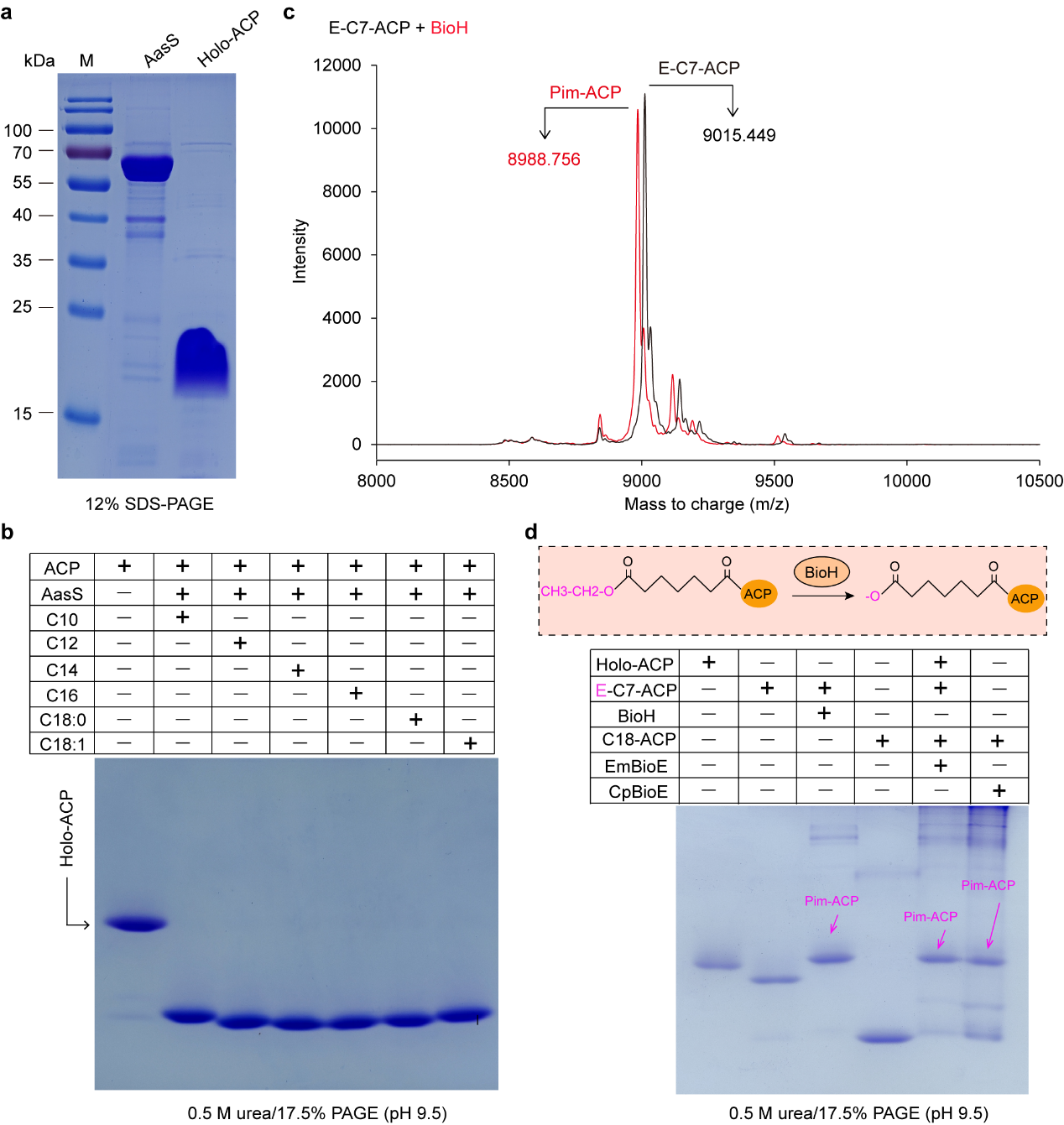


**Figure S7 Generation of diverse chain-length acyl-ACPs using the AasS protein tool**

**a**. SDS-PAGE analysis of purified AasS and Holo-ACP

**b**. Urea gel analysis of acyl-ACPs with different chain lengths

**c**. MALDI-TOF was used to verify the standard molecular weight of C7-ACP, the product of BioH-mediated cleavage of E-C7-ACP.

**d.** BioE catalyzes stearoyl-ACP to produce pimeloyl-ACP

Using C7-ACP generated by BioH-mediated cleavage of E-C7-ACP as a control, both CpBioE and EmBioE were shown to convert stearoyl-ACP into pimeloyl-ACP. The reaction products migrated to the same position as the control on Urea-PAGE. AasS is from *Vibrio harveyi* and Holo-ACP is from *Escherichia coli*.

**Designations:** ACP, Acyl carrier protein; C7-ACP, pimeloyl-ACP; E-C7-ACP, Ethyl-pimeloyl-ACP


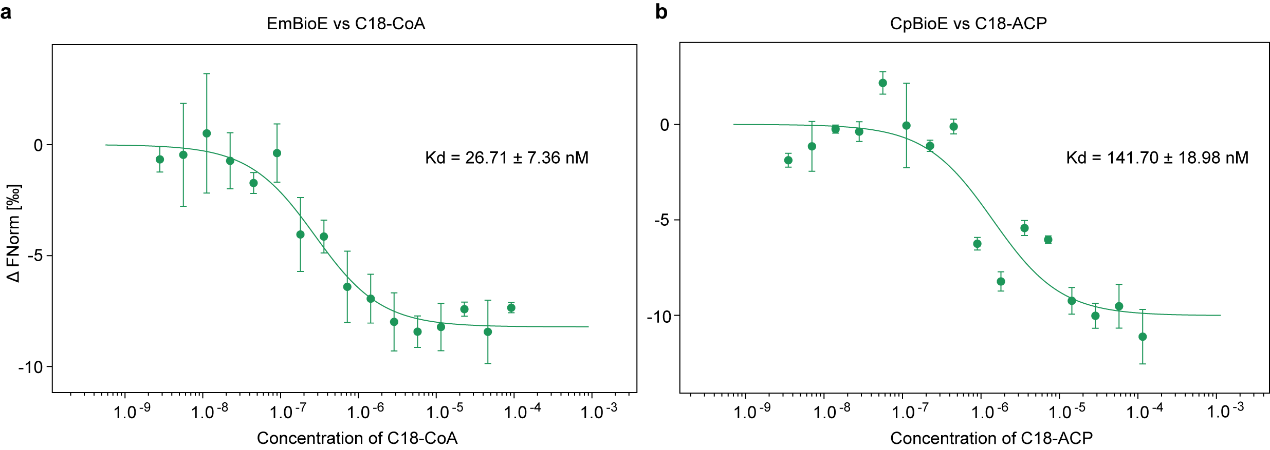


**Figure S8 Microscale Thermophoresis (MST) validates the binding of BioE to long-chain acyl substrates**

**a.** MST confirms that the dissociation constant (Kd) of EmBioE for C18-CoA is approximately 26.71 nM.

**b.** CpBioE has a dissociation constant (Kd) of approximately 141 nM for C18-ACP, determined by MST.

All experiments were performed in three independent replicates (n=3), and values are presented as mean ± SD.


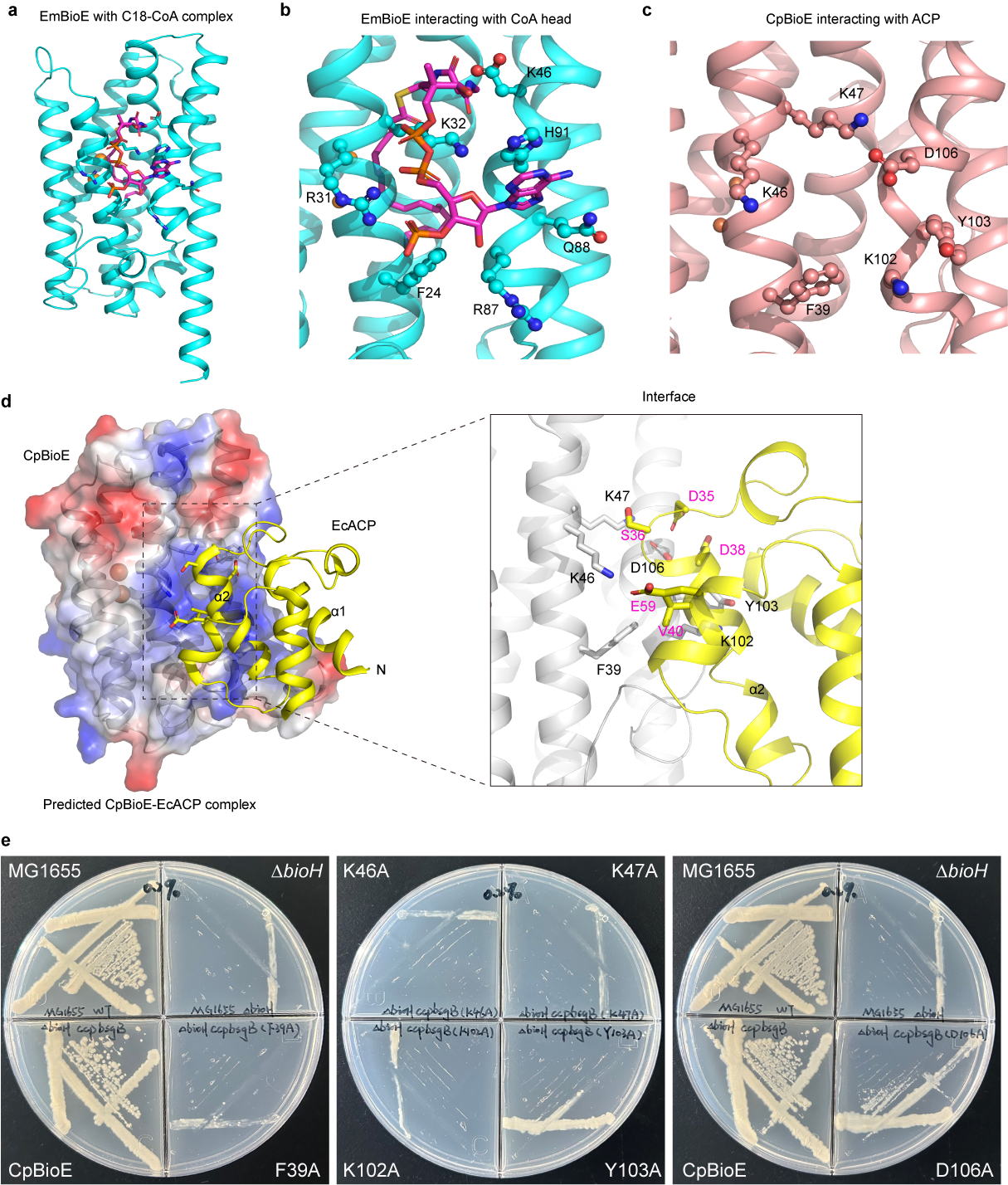


**Figure S9 Prediction of the key residues mediating ACP headgroup binding in CpBioE**

**a.** Molecular docking reveals interactions between EmBioE and C18-CoA

**b**. The residues interacting with CoA headgroup in EmBioE

Residues interacting with the CoA headgroup in EmBioE include F24, R31, K32, K46, R87, Q88, and H91, primarily positively charged residues with cationic side chains.

**c-d**. Putative ACP-interacting region in CpBioE

Structural alignment with EmBioE identified six conserved residues in CpBioE (F39, K46, K47, K102, Y103, and D106) within the predicted ACP-binding interface(**c**). The electrostatic map shows that the negatively charged helix 2 of EcACP forms an interaction with the positively charged region of CpBioE (d). EcACP is derived from the FabB-ACP complex (7SZ9).

**e**. Site-directed mutagenesis confirms functional necessity of ACP-interacting residues

Substitution of F39, K46, K47, K102, Y103, and D106 with alanine in CpBioE abolished growth rescue of *E. coli* MG1655 Δ*bioH* on biotin-free M9 minimal medium. A representative image was chosen from three biological replicates for display.


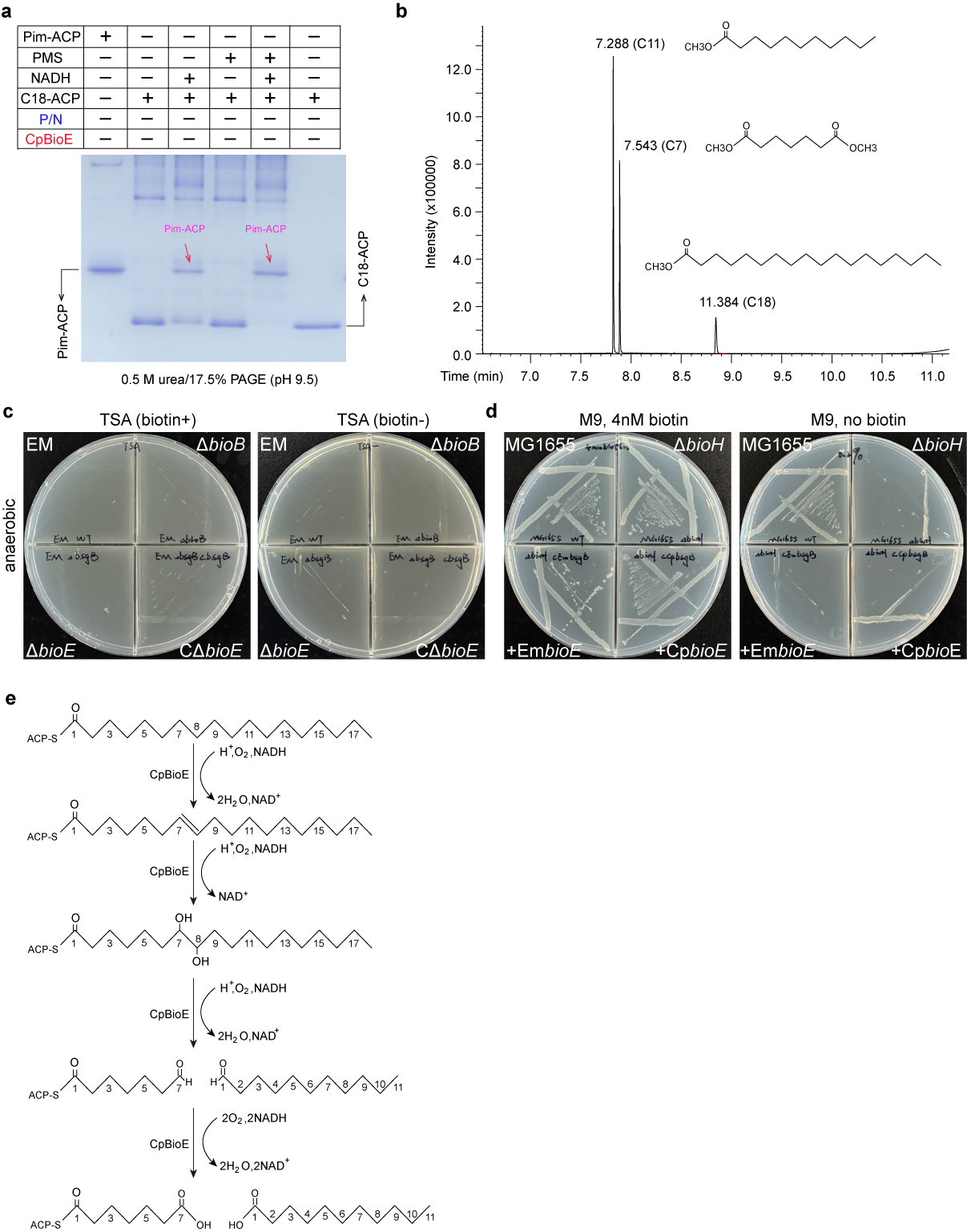


**Figure S10 Proposed mechanism of BioE-catalyzed generation of pimeloyl thioester from long-chain acyl groups**

**a**. CpBioE catalyzes the conversion of C18-ACP to Pim-ACP, which requires the presence of the electron transport system PMS/NADH.

**b**. GC-MS was used to confirm that the two products generated from CpBioE-catalyzed conversion of C18-ACP are C7-ACP and undecanoic acid.

**c-d**. BioE fails to rescue the biotin auxotrophic phenotype of *E. coli bioH* mutants under anaerobic culture conditions.

Under anaerobic conditions, *E. meningoseptica* failed to grow in media with or without biotin, indicating that this bacterium is an obligate aerobe (**c**). For *E. coli* *bioH* mutants complemented with EmBioE or CpBioE, growth was observed under anaerobic conditions in biotin-containing medium but not in biotin-free M9 minimal medium, confirming that the reaction catalyzed by BioE requires oxygen (**d**). One representative image from three biological replicates is shown.

**e**. Proposed reaction process of CpBioE-catalyzed conversion of C18-ACP to C7-ACP and undecanoic acid.

**Designations:** P/N, PMS (Phenazine methosulfate) and NADH (Nicotinamide adenine dinucleotide)


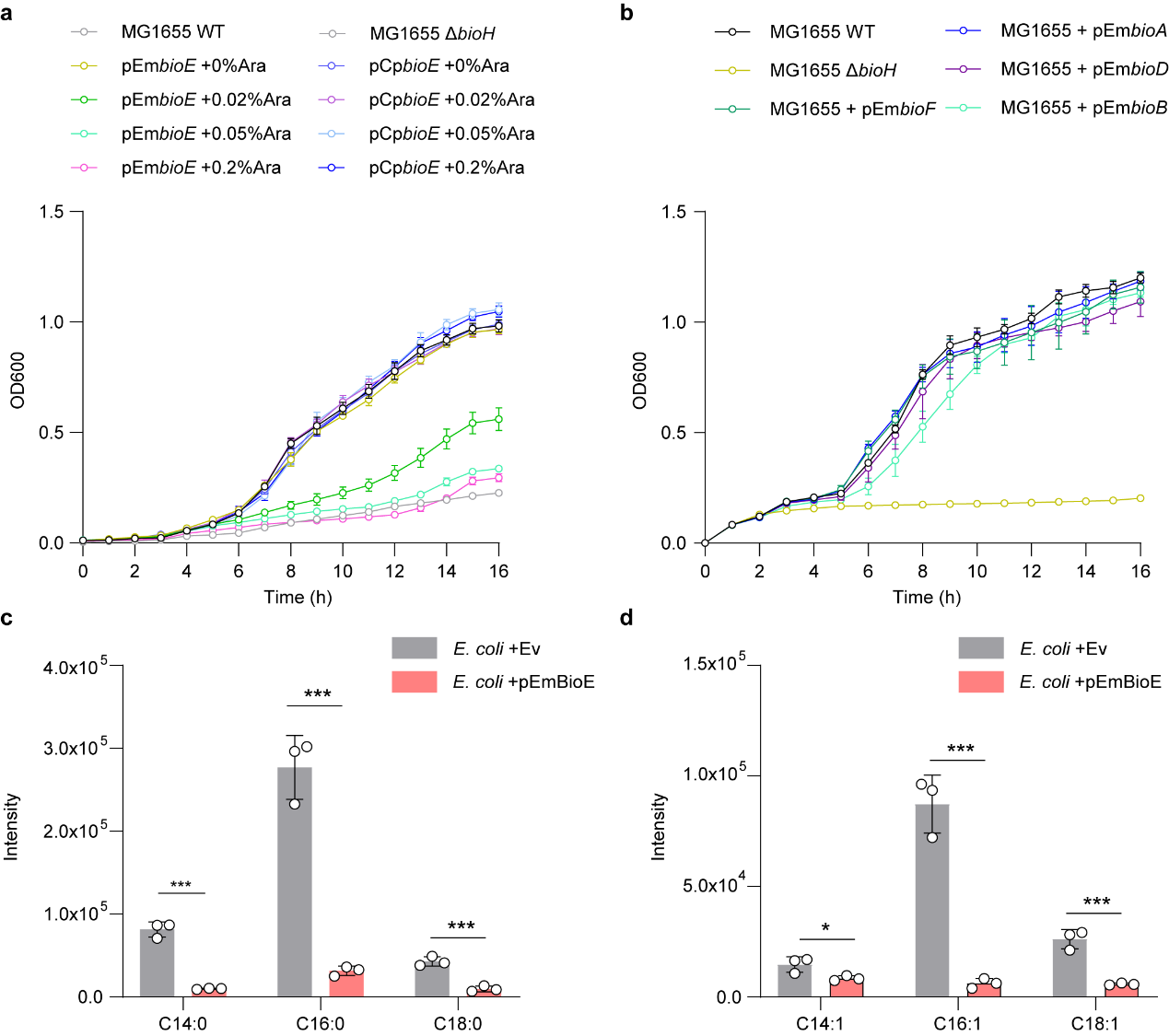


**Figure S11 Overexpression of EmBioE depletes the bacterial long-chain fatty acid pool**

**a**. The growth inhibition of *E. coli* MG1655 by EmBioE is positively correlated with its expression level

Expression of EmBioE and CpBioE was induced with arabinose at different final concentrations (0, 0.02%, 0.05%, and 0.2%). EmBioE-induced growth inhibition of *E. coli* MG1655 was arabinose concentration-dependent, whereas CpBioE had no effect on host growth across all tested induction concentrations.

**b**. Effects of overexpressing late-stage biotin biosynthesis proteins on host bacterial growth

Four proteins (BioF/A/D/B) involved in late-stage biotin synthesis were induced for overexpression with 0.2% arabinose. Only BioB overexpression exerted a mild effect on cell growth; the other proteins had no impact on bacterial growth. The growth curves (**a** and **b**) were presented as the means ± SD, with data derived from three independent experiments.

**c-d**. GC-MS validation that EmBioE overexpression depletes host bacterial long-chain fatty acids

*E. coli* MG1655 strains harboring either the pBAD24 empty vector or pBAD24-EmBioE were analyzed via GC-MS under induction with 0.2% arabinose. The EmBioE-overexpressing strain showed significantly reduced concentrations of saturated long-chain fatty acids (C14:0, C16:0, C18:0) (**c**) and unsaturated long-chain fatty acids (C14:1, C16:1, C18:1) (**d**). The experiment was performed in three independent replicates, and the results are presented as the mean ± SD. Statistical significance was determined using an unpaired two-tailed Student’s t-test (*P < 0.05, ***P < 0.001).


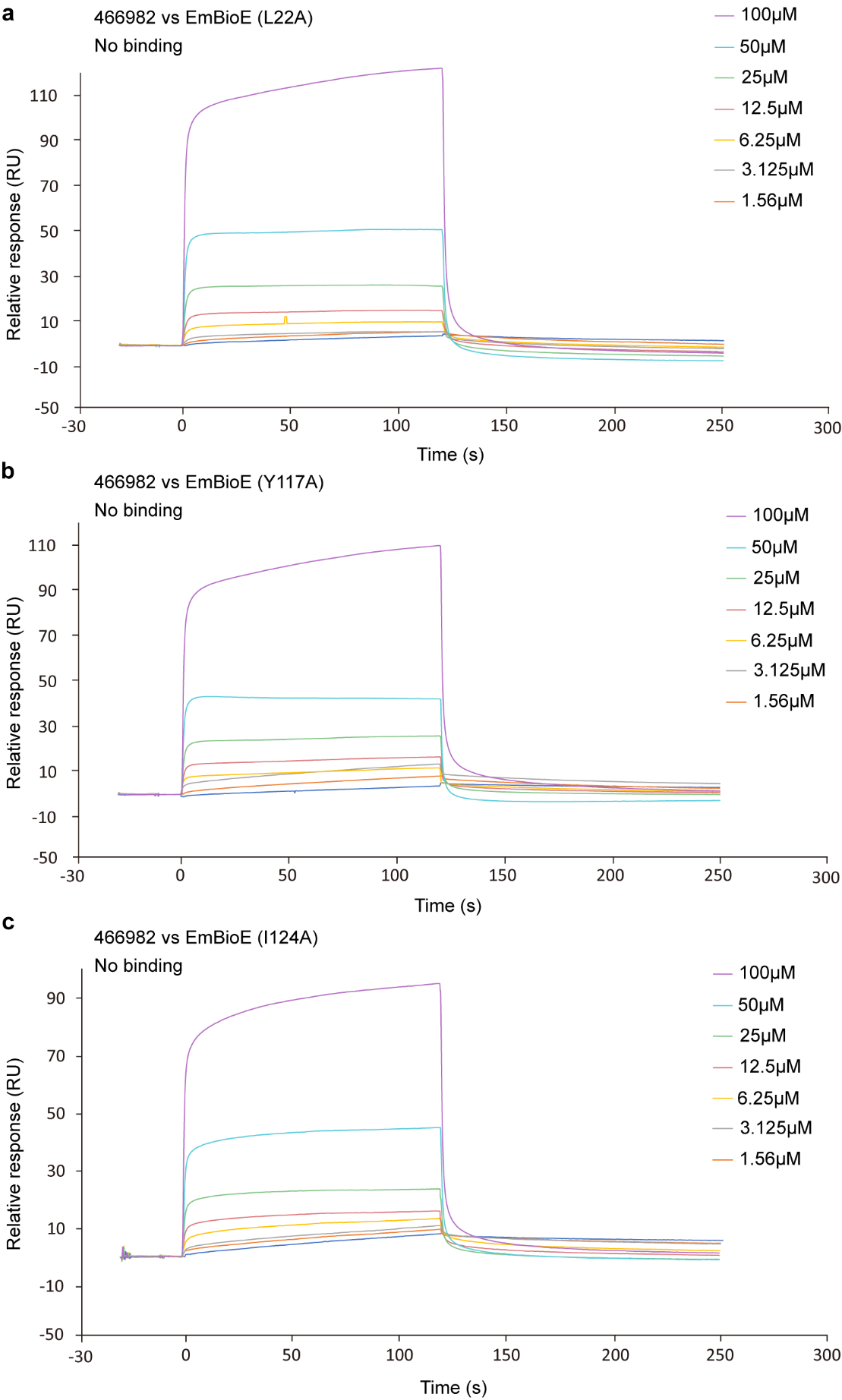


**Figure S12 Mutations of hydrophobic residues result in the loss of their binding to 466982**

Molecular docking results revealed that L22, Y117, and I124 are not only involved in interactions with the long-chain acyl groups of the substrate but also in interactions with substrate 466982. SPR data further demonstrated that the L22A(**a**), Y117A(**b**), and I124A(**c**) mutants fail to bind effectively to the inhibitor 466982. Experiments were performed in three independent replicates, and one representative profile is
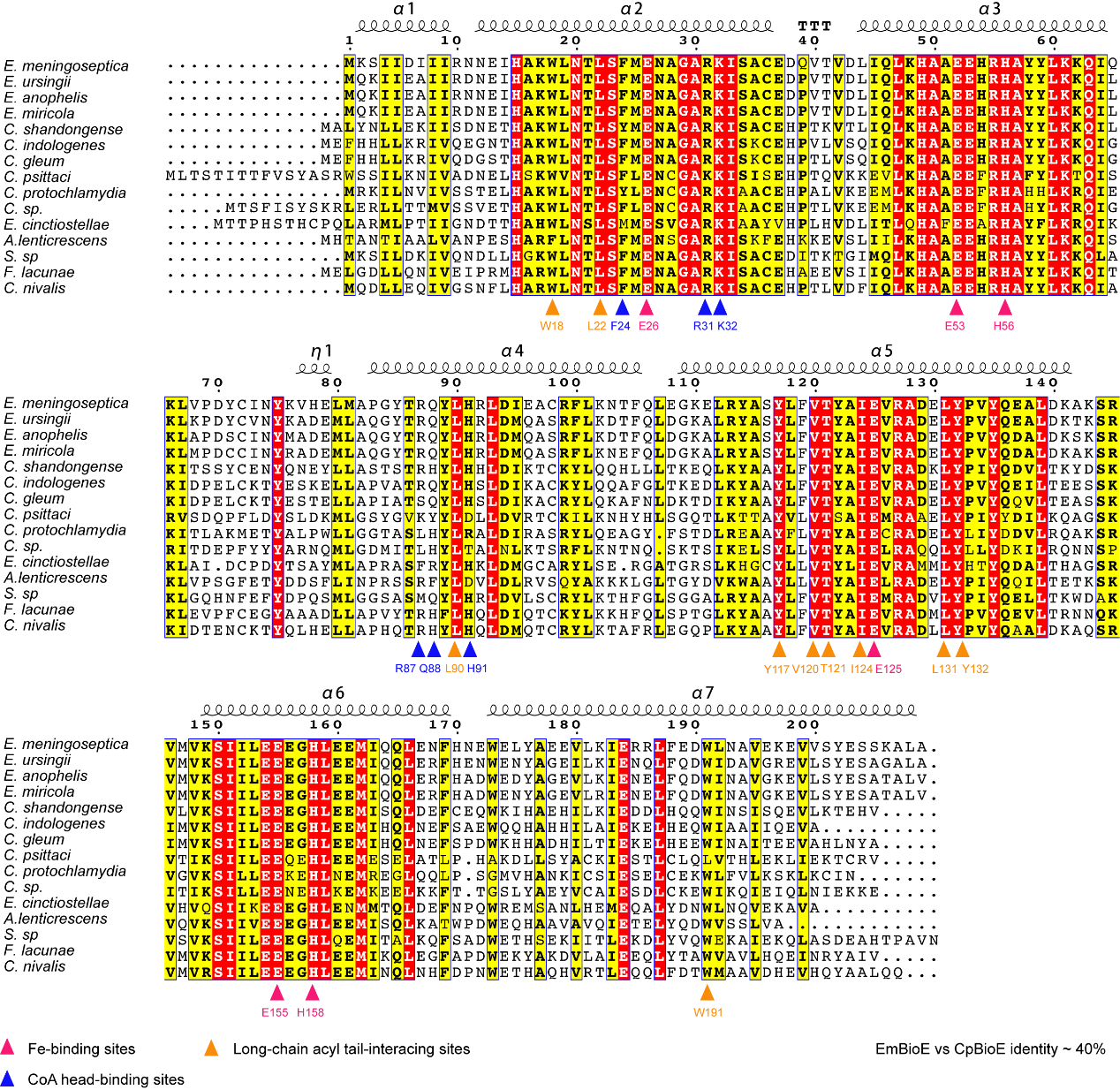
presented.

**Figure S13 Multiple sequence alignment reveals conserved active sites in BioE proteins**

Red triangles indicate amino acids coordinating with two iron atoms; blue triangles denote amino acids interacting with the CoA or ACP headgroup; orange highlights amino acid residues engaging with the hydrophobic long-chain acyl side chain.

**Supporting tables**

**Table S1** Bacterial strains and plasmids used in this study

| **Strains/plasmids** | **Description** | **Origins** |
| --- | --- | --- |
| **Strains** | | |
| DH5α | A cloning host of *E. coli* | Lab stock |
| BL21 | An expression host of *E. coli* | Lab stock |
| DH5α-λpir | A cloning host of pRE112 plasmid | Lab stock |
| *E. coli* K12 MG1655 | A laboratory strain that is easy to genetically manipulate | Lab stock |
| *E. coli* X7213 | Diaminopimelic acid auxotrophic strain, conjugative transfer strain | Lab stock |
| Xu99 | DH5α carrying pBAD322 | This work |
| Xu100 | *Elizabethkingia meningoseptica* EM-1 | This work |
| Xu104 | DH5α-λpir carrying pRE112-Em*bioE* | This work |
| Xu105 | X7213 carrying pRE112-Em*bioE* | This work |
| Xu106 | *E*. *meningoseptica* Δ*bioE* | This work |
| Xu107 | DH5α-λpir carrying pRE112 Em*bioE*+Strep | This work |
| Xu108 | X7213 carrying pRE112 Em*bioE*+Strep | This work |
| Xu109 | *E*. *meningoseptica* C*bioE*+Strep | This work |
| STL24 | MG1655 Δ*bioH* | This work |
| Xu111 | MG1655 Δ*bioH* carrying pBAD24 Em*bioE* | This work |
| Xu1618 | MG1655 Δ*bioH* carrying pBAD24 Cp*bioE* | This work |
| STL96 | MG1655 Δ*bioC* | This work |
| Xu114 | MG1655 Δ*bioC* carrying pBAD24 Em*bioE* | This work |
| Xu1620 | MG1655 Δ*bioC* carrying pBAD24 Cp*bioE* | This work |
| Xu116 | MG1655 Δ*bioC* Δ*bioH* | This work |
| ER90 | A biotin auxotrophic strain of MG1655  (Δ*bioFCD*) | This work |
| Xu117 | MG1655 Δ*bioC* Δ*bioH* carrying pBAD24 Em*bioE* | This work |
| Xu1620 | MG1655 Δ*bioC* Δ*bioH* carrying pBAD24 Cp*bioE* | This work |
| Xu119 | DH5α-λpir carrying pRE112 ΔEm*bioF* | This work |
| Xu120 | X7213 carrying pRE112 ΔEm*bioF* | This work |
| Xu121 | Em Δ*bioF* | This work |
| Xu126 | MG1655 Δ*bioF* Δ*bioH* | This work |
| Xu127 | MG1655 Δ*bioF* Δ*bioH* carrying pBAD24 Em*bioE* | This work |
| Xu128 | MG1655 Δ*bioF* Δ*bioH* carrying pBAD24 Em*bioE* pBAD322 Bs*bioF* | This work |
| Xu153 | *E*. *meningoseptica* Δ*bioL* | This work |
| Xu154 | DH5α-λpir carrying pRE112 Δ*bioL* | This work |
| Xu155 | X7213 carrying pRE112 Δ*bioL* | This work |
| Xu156 | *E*. *meningoseptica* C*bioL-*6xhis | This work |
| Xu157 | DH5α-λpir carrying pRE112 Em*bioL*-6xhis | This work |
| Xu158 | X7213 carrying pRE112 Em*bioL*-6xhis | This work |
| Xu1109 | DH5α-λpir carrying pRE112 CEm*bioL*(R50A) | This work |
| Xu1294 | X7213 carrying pRE112 CEm*bioL*(R50A) | This work |
| Xu1288 | Em C*bioL*(R50A) | This work |
| Xu1634 | DH5α-λpir carrying pRE112 CEm*bioL*（K340A) | This work |
| Xu1635 | X7213 carrying pRE112 CEm*bioL*(K340A) | This work |
| Xu689 | Em C*bioL*(K340A) | This work |
| Xu1636 | BL21 carrying pBAD24 Cp*bioE* | This work |
| Xu1621 | MG1655 Δ*bioH* carrying pBAD24 Cp*bioE*(E41A） | This work |
| Xu1622 | MG1655 Δ*bioH* carrying pBAD24 Cp*bioE*(E68A） | This work |
| Xu1623 | MG1655 Δ*bioH* carrying pBAD24 Cp*bioE*(H71A） | This work |
| Xu1624 | MG1655 Δ*bioH* carrying pBAD24 Cp*bioE*(E140A） | This work |
| Xu1625 | MG1655 Δ*bioH* carrying pBAD24 Cp*bioE*(E170A） | This work |
| Xu1627 | MG1655 carryingpBAD24 Cp*bioE* | This work |
| Xu1341 | MG1655 carrying pBAD24 Em*bioE* | This work |
| Xu1629 | MG1655 carrying pBAD24 Em*bioE*(E26A) | This work |
| Xu1637 | MG1655 carrying pET28a *aasS* | This work |
| Xu1638 | MG1655 carrying pET28a *aasS* pBAD24 Em*bioE* | This work |
| Xu1642 | DH5α-λpir pRE112 ΔEm*bioD* | This work |
| Xu1643 | X7213 pRE112 ΔEm*bioD* | This work |
| Xu1630 | Em Δ*bioD* | This work |
| Xu1631 | Em Δ*bioL* Δ*bioD* | This work |
| Xu816 | MG1655 Δ*bioH* carrying PBAD24 CEm*bioE*(L22A) | This work |
| Xu1633 | MG1655 Δ*bioH* carryingPBAD24 CEm*bioE*(Y117A) | This work |
| Xu820 | MG1655 Δ*bioH* carrying PBAD24 CEm*bioE*(I124A) | This work |
| Xu821 | MG1655 Δ*bioH* carrying PBAD24 CEm*bioE*(L131A) | This work |
| Xu222 | *Chryseobacterium indologenes* Ci | This work |
| Xu223 | *C. indologenes* Δ*bioE* | This work |
| Xu224 | DH5α-λpir carrying pRE112 Rif ΔCi*bioE* | This work |
| Xu225 | X7213 carrying pRE112 Rif ΔCi*bioE* | This work |
| Xu229 | DH5α-λpir carrying pRE112 Rif C*bioE*+strep | This work |
| Xu230 | X7213 carrying PRE112 Rif C*bioE*+strep | This work |
| Xu231 | Ci C*bioE*+strep | This work |
| Xu1644 | MG1655 Δ*bioH* carrying PBAD24 Cp*bioE*（W33A) | This work |
| Xu1645 | MG1655 Δ*bioH* carrying PBAD24 Cp*bioE*（L37A) | This work |
| Xu1646 | MG1655 Δ*bioH* carrying PBAD24 Cp*bioE*（V135A) | This work |
| Xu1647 | MG1655 Δ*bioH* carrying PBAD24 Cp*bioE*（L105A) | This work |
| Xu1648 | MG1655 Δ*bioH* carrying PBAD24 Cp*bioE*（T136A) | This work |
| Xu1649 | MG1655 Δ*bioH* carrying PBAD24 Cp*bioE*（I139A) | This work |
| Xu1650 | MG1655 Δ*bioH* carrying PBAD24 Cp*bioE*（L40A) | This work |
| Xu1651 | MG1655 Δ*bioH* carrying PBAD24 Cp*bioE*（I48A) | This work |
| Xu1652 | MG1655 Δ*bioH* carrying PBAD24 Cp*bioE*（Y150A) | This work |
| Xu1653 | MG1655 Δ*bioH* carrying PBAD24 Cp*bioE*（L146A) | This work |
| Xu1654 | MG1655 Δ*bioH* carrying PBAD24 Cp*bioE*（Y147A) | This work |
| Xu1655 | MG1655 Δ*bioH* carrying PBAD24 Cp*bioE*（L205A) | This work |
| Xu1656 | MG1655 Δ*bioH* carrying PBAD24 Cp*bioE*（F39A) | This work |
| Xu1657 | MG1655 Δ*bioH* carrying PBAD24 Cp*bioE*（K46A) | This work |
| Xu1658 | MG1655 Δ*bioH* carrying PBAD24 Cp*bioE*（K47A) | This work |
| Xu1659 | MG1655 Δ*bioH* carrying PBAD24 Cp*bioE*（K102A) | This work |
| Xu1660 | MG1655 Δ*bioH* carrying PBAD24 Cp*bioE*（Y103A) | This work |
| Xu1661 | MG1655 Δ*bioH* carrying PBAD24 Cp*bioE*（D106A) | This work |
| Xu1644 | MG1655 Δ*bioH* carrying PBAD24 Cp*bioE*（W33A) | This work |
| Xu1645 | MG1655 Δ*bioH* carrying PBAD24 Cp*bioE*（L37A) | This work |
| Xu189 | MG1655 Δ*bioF* | This work |
| Xu190 | MG1655 Δ*bioF* carrying PBAD24 Bs*bioF* | This work |
| Xu191 | MG1655 Δ*bioF* carrying PBAD24 Cp*bioF* | This work |
| Xu192 | MG1655 Δ*bioH* Δ*bioF* carrying Bs*bioF* Bs*bioW* | This work |
| Xu193 | MG1655 Δ*bioH* Δ*bioF* carrying Cp*bioF* Bs*bioW* | This work |
| Xu10 | BL21 carrying pET28a Ec*acpP* pBAD24 Ec*acpS* | This work |
| Xu197 | BL21 carrying pET28a Em*bioE* | This work |
| Xu232 | BL21 carrying pGEX-6P-1 carrying sumo Em*bioL* | This work |
| Xu876 | MG1655 carrying pGEN-luxCDAE p*bioE* pBAD24 CEm*bioL* | This work |
| Xu877 | MG1655 carrying pGEN-luxCDAE p*bioB* pBAD24 CEm*bioL* | This work |
| **Plasmids** |  |  |
| pET21a | a T7 promoter-driven expression vector, Amp^R^ | Lab stock |
| pET28a | a T7 promoter-driven expression vector, Km^R^ | Lab stock |
| pBAD | an arabinose-induced expression vector, Amp^R^ | Lab stock |
| pBAD24*::*Em*bioE* | pBAD24 encoding CEm*bioE* AmpR | This work |
| pBAD24*::*Cp*bioE* | pBAD24 encoding Cp*bioE* Amp^R^ | This work |
| pRE112 ErmF*::*ΔEm  *bioD* Up+Down | pRE112 encoding ΔEm*bioD* Up+Down Chl^R^ | This work |
| pRE112 ErmF*::*ΔEm  *bioE* Up+Down | pRE112 encoding ΔEm*bioE* Up+Down Chl^R^ | This work |
| pRE112 ErmF*::*ΔEm  *bioL* Up+Down | pRE112 encoding ΔEm*bioL* Up+Down Chl^R^ | This work |
| pRE112 ErmF*::*CEm  *bioL* EmΔ*bioL* Up+Down | pRE112 encoding CEm*bioL* EmΔ*bioL* Up+Down Chl^R^ | This work |
| pRE112 ErmF*::*C*bioL*  (R50A) EmΔ*bioL* Up+Down | pRE112 encoding the mutant version of EmΔ*bioL* (R50A) Up+Down Chl^R^ | This work |
| pRE112 ErmF*::*C*bioL*  (K340A) EmΔ*bioL* Up+Down | pRE112 encoding the mutant version of EmΔ*bioL* (K340A) Up+Down Chl^R^ | This work |
| pET28a*::*Em*bioE* | pET28a encoding Em*bioE* Km^R^ | This work |
| pBAD24*::*Cp*bioE*(E41A) | pBAD24 encoding the mutant version of Cp*bioE*(E41A) Amp^R^ | This work |
| pBAD24*::*Cp*bioE*(E68A) | pBAD24 encoding the mutant version of Cp*bioE*(E68A) Amp^R^ | This work |
| pBAD24*::*Cp*bioE*(H71A) | pBAD24 encoding the mutant version of Cp*bioE*(H71A) Amp^R^ | This work |
| pBAD24*::*Cp*bioE*(E140A) | pBAD24 encoding the mutant version of Cp*bioE*(E140A) Amp^R^ | This work |
| pBAD24*::*Cp*bioE*(E170A) | pBAD24 encoding the mutant version of Cp*bioE*(E170A) Amp^R^ | This work |
| pBAD24*::*Cp*bioE*(H173A) | pBAD24 encoding the mutant version of Cp*bioE*(H173A) Amp^R^ | This work |
| pBAD24*::*Em*bioE*(E26A) | pBAD24 encoding the mutant version of Em*bioE*(E26A) Amp^R^ | This work |
| pET28a*::aasS* | pET28a encoding aasS Km^R^ | This work |
| pBAD24*::*Em*bioE*(L22A) | pBAD24 encoding the mutant version of Em*bioE*(L22A) Amp^R^ | This work |
| pBAD24*::*Em*bioE*(Y117A) | pBAD24 encoding the mutant version of Em*bioE*(Y117A) Amp^R^ | This work |
| pBAD24*::*Em*bioE*(I124A) | pBAD24 encoding the mutant version of Em*bioE*(I124A) Amp^R^ | This work |
| pBAD24*::*Em*bioE*(L131A) | pBAD24 encoding the mutant version of Em*bioE*(L131A) Amp^R^ | This work |
| pRE112 Rif9*::*ΔCi*bioE* Up+Down | pRE112 Rif encoding ΔCi*bioE* Up+Down Chl^R^ | This work |
| pRE112 Rif*::*CCi*bioE* CiΔ*bioE* Up+Down | pRE112 Rif encoding C*bioE* EmΔ*bioE* Up+Down Chl^R^ | This work |
| pBAD24*::*Cp*bioE*(W33A) | pBAD24 encoding the mutant version of Cp*bioE*(W33A) Amp^R^ | This work |
| pBAD24*::*Cp*bioE*(L37A) | pBAD24 encoding the mutant version of Cp*bioE*(L37A) Amp^R^ | This work |
| pBAD24*::*Cp*bioE*(V135A) | pBAD24 encoding the mutant version of Cp*bioE*(V135A) Amp^R^ | This work |
| pBAD24*::*Cp*bioE*(L105A) | pBAD24 encoding the mutant version of Cp*bioE*(L105A) Amp^R^ | This work |
| pBAD24*::*Cp*bioE*(T136A) | pBAD24 encoding the mutant version of Cp*bioE*(T136A) Amp^R^ | This work |
| pBAD24*::*Cp*bioE*(I139A) | pBAD24 encoding the mutant version of Cp*bioE*(I139A) Amp^R^ | This work |
| pBAD24*::*Cp*bioE*(L40A) | pBAD24 encoding the mutant version of Cp*bioE*(L40A) Amp^R^ | This work |
| pBAD24*::*Cp*bioE*(I48A) | pBAD24 encoding the mutant version of Cp*bioE*(I48A) Amp^R^ | This work |
| pBAD24*::*Cp*bioE*(Y150A) | pBAD24 encoding the mutant version of Cp*bioE*(Y150A) Amp^R^ | This work |
| pBAD24*::*Cp*bioE*(L146A) | pBAD24 encoding the mutant version of Cp*bioE*(L146A) Amp^R^ | This work |
| pBAD24*::*Cp*bioE*(Y147A) | pBAD24 encoding the mutant version of Cp*bioE*(Y147A) Amp^R^ | This work |
| pBAD24*::*Cp*bioE*(L205A) | pBAD24 encoding the mutant version of Cp*bioE*(L205A) Amp^R^ | This work |
| pBAD24*::*Cp*bioE*(F39A) | pBAD24 encoding the mutant version of Cp*bioE*(F39A) Amp^R^ | This work |
| pBAD24*::*Cp*bioE*(K46A) | pBAD24 encoding the mutant version of Cp*bioE*(K46A) Amp^R^ | This work |
| pBAD24*::*Cp*bioE*(K47A) | pBAD24 encoding the mutant version of Cp*bioE*(K47A) Amp^R^ | This work |
| pBAD24*::*Cp*bioE*(K102A) | pBAD24 encoding the mutant version of Cp*bioE*(K102A) Amp^R^ | This work |
| pBAD24*::*Cp*bioE*(Y103A) | pBAD24 encoding the mutant version of Cp*bioE*(Y103A) Amp^R^ | This work |
| pBAD24*::*Cp*bioE*(D106A) | pBAD24 encoding the mutant version of Cp*bioE*(D106A) Amp^R^ | This work |
| pBAD24*::*CBs*bioF* | pBAD24 encoding Bs*bioF* Amp^R^ | This work |
| pBAD24*::*CCp*bioF* | pBAD24 encoding Cp*bioF* Amp^R^ | This work |
| pGEX-6P-1*::*sumo Em*bioL* | pGEX-6P-1 encoding sumo Em*bioL* Amp^R^ | This work |
| pGEN-luxCDAE*::*p*bioE* | pGEN-luxCDAE encoding p*bioE* Amp^R^ | This work |
| pGEN-luxCDAE*::*pbioB | pGEN-luxCDAE encoding pbioB Amp^R^ | This work |
| pBAD24*::*CEm*bioL* | pBAD24 encoding CEm*bioL* Chl^R^ | This work |
| pET28a*::*Ec*acpP* | pET28a*::* encoding Ec*acpP* Km^R^ | This work |
| pBAD24*::*Ec*acpS* | pBAD24*::* encoding Ec*acpS* Amp^R^ | This work |

**Table S2** Primers used in this study

| **Primers** | **Sequences** |
| --- | --- |
| ΔEm*bioE*-Up-F | 5'GTAAGTGAACTGCATGAATTCCCGGGGAAATTCAAAATCATAATCATC-3' |
| ΔEm*bioE*-Up-R | 5'-CCAGTGCTTTGGAAGATTCATCTCTTTTTTGAAGCAAAATTATTATC-3' |
| ΔEm*bioE*-Down-F | 5'-GATAATAATTTTGCTTCAAAAAAGAGATGAATCTTCCAAAGCACTG  G-3' |
| ΔEm*bioE*-Down-R | 5'-GGAAAGTGGTAAAGGTACCGCATGCACATCACGGGATAAGCATTT  AT-3' |
| Em*bioE*+Strep-Up-F | 5'-TGAACTGCATGAATTCCCGGGATGAAAAGTATAATTGATATTATT  ATC-3' |
| Em*bioE*+Strep-Up-R | 5'-TTATTTTTCGAACTGCGGGTGGCTCCACGCCAGTGCTTTGGAA  GATTC-3' |
| Em*bioE*+Strep-Down-F | 5'-TGGAGCCACCCGCAGTTCGAAAAATAAATGAATCTTCCAAAGC  ACTGG-3' |
| Em*bioE*+Strep-Down-R | 5'-ACTGGAAAGTGGTAAAGGTACCTTAGTATTCATTCTGATTTTTTT  TAA-3' |
| pBAD24-Em*bioE*-F | 5'-GAGGAATTCACCATGGTACCCGGGATGAAAAGTATAATTGATATTA  TT-3' |
| pBAD24-Em*bioE*-R | 5'-GCCAAAACAGCCAAGCTTGCATGCTTACGCCAGTGCTTTGGAA  GAT-3' |
| pBAD24-Cp*bioE*-F | 5'-GAGGAATTCACCATGGTACCCGGGATGCTAACTTCAACAATAA  CGAC-3' |
| pBAD24-Cp*bioE*-R | 5'-GCCAAAACAGCCAAGCTTGCATGCTTACACACGACATGTCTTTT  CAAT-3’ |
| Em*bioE*(L22A)-F | 5'-CTCAATACCGCATCGTTTATGGAAAATGCAGGTG-3' |
| Em*bioE*(L22A)-R | 5'-AACGATGCGGTATTGAGCCACTTCGCGTGTAT-3' |
| Em*bioE*(E26A)-F | 5'-TATGGCAAATGCAGGTGCCCGTAAAATTTCAG-3' |
| Em*bioE*(E26A)-R | 5'-CACCTGCATTTGCCATAAACGAAAGGGTATTGAGCC-3' |
| Em*bioE*(Y117A)-F | 5'-GAGGAATTCACCATGGTACCCGGGATGAATCTTCCAAAGCACT  GGC-3' |
| Em*bioE*(Y117A)-R | 5'-CAAACAGTGCGGAAGCATAACGCAATTCTTTCC-3' |
| Em*bioE*(I124A)-F | 5'-CTATGCCGCAGAAGTAAGAGCAGACGAATTATATCCC-3' |
| Em*bioE*(I124A)-R | 5'-TTACTTCTGCGGCATAGGTAACAAACAGATAGGAAG-3' |
| Em*bioE*(L131A)-F | 5'-AGACGAAGCATATCCCGTATATCAGGAAGCATTG-3' |
| Em*bioE*(L131A)-R | 5'-CGGGATATGCTTCGTCTGCTCTTACTTCTATGGC-3' |
| qEm*bioE*-F | 5'-GCGAAGTGGCTCAATACCCT-3' |
| qEm*bioE*-R | 5'-GGCTTCAATATCCAGCCGGT-3' |
| qEm*bioD*-F | 5'-GGTATCGGAACCGGAATCGG-3' |
| qEm*bioD*-R | 5'-CTGATGTGGTGATGCGGGAT-3' |
| M13-F | 5'-TGTAAAACGACGGCCAGT-3' |
| M13-R | 5'-CAGGAAACAGCTATGACC-3' |
| probe-P*bioE*-F | 5'-TTTTAAATCTGGTACCATTTAAGGTCCGGCAAGATAATAATTT  TGCTTCAAAAAAGAG-3' |
| probe-P*bioE*-R | 5'-CTCTTTTTTGAAGCAAAATTATTATCTTGCCGGACCTTAAATGGTAC  CAGATTTAAAA-3' |
| probe-P*bioB*-F | 5'-TGCTTTTATCAAACTCATGTGTAATTACTCTGGTAATCTATAT  GATTAAGATTTAGAAG-3' |
| probe-P*bioB*-R | 5'-CTTCTAAATCTTAATCATATAGATTACCAGAGTAATTACACATGAGTT  TGATAAAAGCA-3' |
| Cp*bioE*(E41A)-F | 5'-CCCTGTCCTTTTTAGCAAATAGTGGAGCAAAAAAAATCTCCG-3' |
| Cp*bioE*(E41A)-R | 5'-TGCTAAAAAGGACAGGGTATTTATCCATTGTG-3' |
| Cp*bioE*(E68A)-F | 5'-TGCTGAAGCATTTCGTCATGGTCACTATCTAAAAACT-3' |
| Cp*bioE*(E68A)-R | 5'-GACGAAATGCTTCAGCAGCATGTTTTAAAACTTCT-3' |
| Cp*bioE*(H71A)-F | 5'-ATTTCGTGCAGGTCACTATCTAAAAACTCAGATTTCTAGA-3' |
| Cp*bioE*(H71A)-R | 5'-AGTGACCTGCACGAAATTCTTCAGCAGCATGTT-3' |
| Cp*bioE*(E140A)-F | 5'-ACGCAATCGCACTTCGTGCTTCTGAACTTTATCCTC-3' |
| Cp*bioE*(E140A)-R | 5'-ACGAAGTGCGATTGCGTAGGTAACTAAAATATACGC-3' |
| Cp*bioE*(E170A)-F | 5'-CTTAGAAGCACAAGGCCATCTGCAAGAGATGG-3' |
| Cp*bioE*(E170A)-R | 5'-GGCCTTGTGCTTCTAAGATAATGGATTTTACCGTTATTT-3' |
| Cp*bioE*(H173A)-F | 5'-TAGAAGAGCAAGGCGCACTGCAAGAGATGGAACGTGAACT-3' |
| Cp*bioE*(H173A)-R | 5'-TGCGCCTTGCTCTTCTAAGATAATGGATTTTA-3' |
| Cp*bioE*(F39A)-F | 5'-CCTGTCCGCATTAGAAAATAGTGGAGCAAAAAAAATC-3' |
| Cp*bioE*(F39A)-R | 5'-TTTCTAATGCGGACAGGGTATTTATCCATTGTGC-3' |
| Cp*bioE*(K46A)-F | 5'-GTGGAGCAGCAAAAATCTCCGCAAGTGAACATCC-3' |
| Cp*bioE*(K46A)-R | 5'-GATTTTTGCTGCTCCACTATTTTCTAAAAAGGACA-3' |
| Cp*bioE*(K47A)-F | 5'-AGCAAAAGCAATCTCCGCAAGTGAACATCCTAC-3' |
| Cp*bioE*(K47A)-R | 5'-CGGAGATTGCTTTTGCTCCACTATTTTCTAAAAAGG-3' |
| Cp*bioE*(K102A)-F | 5'-GAGGCTTACTTACAGCATATTACCTCCATCTTCTAGATTTAAGGA  CG-3' |
| Cp*bioE*(K102A)-R | 5'-TGCTGTAAGTAAGCCTCCCAGAAGATTTTTAG-3' |
| Cp*bioE*(Y103A)-F | 5'-AGCATACCTCCATCTTCTAGATTTAAGGACGT-3' |
| Cp*bioE*(Y103A)-R | 5'-GAAGATGGAGGTATGCTTTTGTAAGTAAGCCTCCCAGAAGA-3' |
| Cp*bioE*(H106A)-F | 5'-TTACCTCGCACTTCTAGATTTAAGGACGTGCCG-3' |
| Cp*bioE*(H106A)-R | 5'-CTAGAAGTGCGAGGTAATATTTTGTAAGTAAGCCTCCC-3' |
| Cp*bioE*(W33A)-F | 5'-GGCATGCACAAGCAATAAATACCCTGTCCTTTTTAGAAAATAGT-3' |
| Cp*bioE*(W33A)-R | 5'-TATTGCTTGTGCATGCCAATAGTTGGAAGCTA-3' |
| Cp*bioE*(L37A)-F | 5'-GGATAAATACCGCATCCTTTTTAGAAAATAGTGGAGCAAA-3' |
| Cp*bioE*(L37A)-R | 5'-GGATGCGGTATTTATCCATTGTGCATGCCAAT-3' |
| Cp*bioE*(L105A)-F | 5'-TTACGCACATCTTCTAGATTTAAGGACGTGCC-3' |
| Cp*bioE*(L105A)-R | 5'-CTAGAAGATGTGCGTAATATTTTGTAAGTAAGCCTCCCAGA-3' |
| Cp*bioE*(V135A)-F | 5'-TAGCAACCTACGCAATCGAACTTCGTGCTTCT-3' |
| Cp*bioE*(L37A)-R | 5'-CCAGAAAAGATGCGGTATTAACCCACTTGCTATGCAG-3' |
| Cp*bioE*(L105A)-F | 5'-ATACTACGCAGACCTGTTGGACATGCGTACCT-3' |
| Cp*bioE*(L105A)-R | 5'-ACAGGTCTGCGTAGTATTTGACGCCGTAAGAGCC-3' |
| Cp*bioE*(V135A)-F | 5'-TATGTGCTGGCAACGAGCGCGATCGAGATGCG-3' |
| Cp*bioE*(V135A)-R | 5'-CTCGTTGCCAGCACATATGCGGTGGTCTTCAG-3' |
| Cp*bioE*(T136A)-F | 5'-TATGTGCTGGTGGCAAGCGCGATCGAGATGCGT-3' |
| Cp*bioE*(T136A)-R | 5'-CTTGCCACCAGCACATATGCGGTGGTCTTCAG-3' |
| Cp*bioE*(I139A)-F | 5'-ACGAGCGCGGCAGAGATGCGTGCAGCTGAGCT-3' |
| Cp*bioE*(I139A)-R | 5'-ATCTCTGCCGCGCTCGTCACCAGCACATATGC-3' |
| Cp*bioE*(L146A)-F | 5'-CTGAGGCATACCCGATTTACCATGACATCTTG-3' |
| Cp*bioE*(L146A)-R | 5'-AATCGGGTATGCCTCAGCTGCACGCATCTCGA-3' |
| Cp*bioE*(Y147A)-F | 5'-TGAGCTGGCACCGATTTACCATGACATCTTGAAG-3' |
| Cp*bioE*(Y147A)-R | 5'-AAATCGGTGCCAGCTCAGCTGCACGCATCTCG-3' |
| Cp*bioE*(L205A)-F | 5'-CTGCAGGCAGTTACTCACCTTGAGAAACTGATTGAA-3' |
| Cp*bioE*(L205A)-R | 5'-TGAGTAACTGCCTGCAGGCAAAGGGTGGACTC-3' |
| pBAD-Em*bioF*-F | 5'-GGAATTCACCATGGTACCCGGGATGAATCTTCCAAAGCACT  GG-3' |
| pBAD-Em*bioF*-R | 5'-CCAAAACAGCCAAGCTTGCATGCCGGTTCCGATACCTGTAAT  AA-3' |
| pBAD-Cp*bioF*-F | 5'-GAGGAATTCACCATGGTACCCGGGATGCCCATAGGAAATCT  ATTAGA-3' |
| pBAD-Cp*bioF*-R | 5'-GCCAAAACAGCCAAGCTTGCATGCTCACTCGAGTTAAGAGA  GGATGCA-3' |
| pBAD-Bs*bioF*-F | 5'-GGAATTCACCATGGTACCCGGGATGTTGAAGATTGATTCCTG  GTT-3' |
| pBAD-Bs*bioF*-R | 5'-GGAATTCACCATGGTACCCGGGCTGTTGCTGTACAATATCA  GCA-3' |
| pBAD-Bs*bioW*-F | 5'-AGGAGGAATTCACCATGGTACCCGGGATGCAAGAAGAAACTTTTT  ATAGTG-3' |
| pBAD-Bs*bioW*-R | 5'-CCGCCAAAACAGCCAAGCTTGCATGCTCATGAGTCATGATCTTCC  TCC-3' |
| pGEN-pEm*bioE*-F | 5'-TTCCTGCAGGGCATGCCCCGGGAGGAATAGTTTTCACCCATC  CTTG-3' |
| pGEN-pEm*bioE*-R | 5'-GTCGACTCTAGAGGATCCCCGGGCTCTTTTTTGAAGCAAAATTA  TTATC-3' |
| pGEN-pEm*bioB*-F | 5'-AATTCCTGCAGGGCATGCCCCGGGGATTTAGAAATGTTATAAAG  TTGTTA-3' |
| pGEN-pEm*bioB*-R | 5'-GTCGACTCTAGAGGATCCCCGGGAAATATTTGATTTTTACTTTTA  ATTTTTT-3' |
| pBAD-Em*bioL*-F | 5’-AGTGAACTGCATGAATTCCCGGGATGGATAGTCCAGTAAAAA  TAC-3’ |
| pBAD-Em*bioL*-R | 5’-CTGGAAAGTGGTAAAGGTACCTTATTTTTCGAACTGCGGGTGG-3’ |
| pET28a-Em*bioE*-F | 5'-GGTGCCGCGCGGCAGCCATATGAAAAGTATAATTGATATTATTA  TCC-3' |
| pET28a-Em*bioE*-R | 5'-GTGGTGGTGGTGGTGGTGCTCGAGTTACGCCAGTGCTTTGGA  AGATTC-3' |
| Sumo-*bioL*-F | 5'-CACAGAGAACAGATTGGTGGATCCATGAGTAGTCCGGATCAG  TTTTTA-3' |
| Sumo-*bioL*-R | 5'-GGTGGTGGTGGTGGTGCTCGAGCTATTTAAGTGATTCATTTAC  CTGA |
| pGEX-Sumo-*bioL*-F | 5'-ATGTCGGACTCAGAAGTCAATCCTGTTCCAGGGGCCCCTGGG  ATCC |
| pGEX-Sumo-*bioL*-R | 5'-TGCGGCCGCTCGAGTCGACCCGGGCTATTTAAGTGATTCATTT  ACCTGA |
| CEm*bioL*(R50A)-F | 5'-GAACCGCAAGCATGAGTGATATTCTGAAGGTACAC-3' |
| CEm*bioL*(R50A)-R | 5'-ACTCATGCTTGCGGTTCCCAGTAATTTCATTCCCT-3' |
| CEm*bioL*(K340A)-F | 5'-TCTTTCGGTGCATCCCTGGCTCCCGGGTTCCG-3' |
| CEm*bioL*(K340A)-R | 5'-AGGGATGCACCGAAAGAACCTACGTAAATTACC-3' |
| CCi*bioE*-Up-F | 5'-AAGTGAACTGCATGAATTCCCGGGAGAGACTTTCCAAACGAA  CCGA-3' |
| CCi*bioE*-Up（strep）-R | 5'-TTATTTTTCGAACTGCGGGTGGCTCCATGCTACTTCCTGAATG  ATTGCG-3' |
| CCi*bioE*-Down（strep）-F | 5'-TGGAGCCACCCGCAGTTCGAAAAATAATTATCCATGCTTATTCA  TCATA-3' |
| CCi*bioE*-Down-R | 5'-TTCTGTCCAGGTTCCGTGGTACCTTGGGCTATAAACGGCATAG  GT-3' |
| ΔCi*bioE*-Up-F | 5'-AAGTGAACTGCATGAATTCCCGGGGGGAGAAAGAAGGATTTCG  GAT-3' |
| ΔCi*bioE*-Up-R | 5'-GAATTTATGATGAATAAGCATGGAGTAGTATATTGCCGGATGGCTA-3' |
| ΔCi*bioE*-Down-F | 5'-TAGCCATCCGGCAATATACTACTCCATGCTTATTCATCATAAATTC-3' |
| ΔCi*bioE*-Down-R | 5'-TCTGTCCAGGTTCCGTGGTACCCATTACACAAGATAGCAGCTCC-3' |
| pET28a-Ec*acpP*-F | 5'-CTTTAAGAAGGAGATATACCATGAGCACTATCGAAGAACGCGT-3' |
| pET28a-Ec*acpP*-R | 5'-GTGGTGGTGGTGGTGGTGCTCGAGTTACGCCTGGTGGCCGTTG  AT-3' |
| pBAD-Ec*acpS*-F | 5'-GAGGAATTCACCATGGTACCCGGGATGGCAATATTAGGTTTAGGC-3' |
| pBAD-Ec*acpS*-R | 5'-GCCAAAACAGCCAAGCTTGCATGCTTAACTTTCAATAATTACCGTG-3' |
| pET28a-*aasS*-F | 5'-CCGCGCGGCAGCCATATGGGATCCATGAACCAGTATGTAAATG  ATCCA-3' |
| pET28a-*aasS*-R | 5'-GTGGTGGTGGTGGTGGTGCTCGAGTTACAGATGAAGTTTAC  GCAGTTC-3' |
